# *Mycobacterium tuberculosis* inhibits the NLRP3 inflammasome activation via its phosphokinase PknF

**DOI:** 10.1371/journal.ppat.1009712

**Published:** 2021-07-29

**Authors:** Shivangi Rastogi, Sarah Ellinwood, Jacques Augenstreich, Katrin D. Mayer-Barber, Volker Briken

**Affiliations:** 1 Department of Cell Biology and Molecular Genetics, University of Maryland, College Park, Maryland, United States of America; 2 Inflammation and Innate Immunity Unit, Laboratory of Clinical Immunology and Microbiology, National Institute of Allergy and Infectious Diseases, National Institutes of Health, Bethesda, Maryland, United States of America; McGill UniversityHealth Centre, CANADA

## Abstract

*Mycobacterium tuberculosis* (Mtb) has evolved to evade host innate immunity by interfering with macrophage functions. Interleukin-1β (IL-1β) is secreted by macrophages after the activation of the inflammasome complex and is crucial for host defense against Mtb infections. We have previously shown that Mtb is able to inhibit activation of the AIM2 inflammasome and subsequent pyroptosis. Here we show that Mtb is also able to inhibit host cell NLRP3 inflammasome activation and pyroptosis. We identified the serine/threonine kinase PknF as one protein of Mtb involved in the NLRP3 inflammasome inhibition, since the *pknF* deletion mutant of Mtb induces increased production of IL-1β in bone marrow-derived macrophages (BMDMs). The increased production of IL-1β was dependent on NLRP3, the adaptor protein ASC and the protease caspase-1, as revealed by studies performed in gene-deficient BMDMs. Additionally, infection of BMDMs with the *pknF* deletion mutant resulted in increased pyroptosis, while the IL-6 production remained unchanged compared to Mtb-infected cells, suggesting that the mutant did not affect the priming step of inflammasome activation. In contrast, the activation step was affected since potassium efflux, chloride efflux and the generation of reactive oxygen species played a significant role in inflammasome activation and subsequent pyroptosis mediated by the Mtb *pknF* mutant strain. In conclusion, we reveal here that the serine/threonine kinase PknF of Mtb plays an important role in innate immune evasion through inhibition of the NLRP3 inflammasome.

## Author summary

*Mycobacterium tuberculosis* (Mtb) infections are causing millions of deaths per year and the pathogen is highly adapted to its human host. Host cell phagocytes take up Mtb but the bacterium is capable of manipulating the host cell to enhance its own survival. In the current study we discover a novel pathway of host cell manipulation and innate immune evasion by Mtb. We show that the activation of a host cell defense complex, the inflammasome, is limited after Mtb infection. Most importantly, we identify a bacterial protein, PknF, that is involved in inflammasome inhibition.

## Introduction

IL-1β is of great importance for host resistance against infections with Mtb [1]. Mouse studies demonstrate the hyper susceptibility of mice deficient in the expression of either IL-1α or IL-1β or the IL1R1- receptor [2–6]. Cell-autonomous mechanisms of bacterial control by IL-1β have been proposed to involve cell intrinsic mechanisms via the increase in host cell apoptosis [7], autophagy signaling [8] or enhanced maturation of Mtb-containing phagosomes and thus limiting bacterial growth [9,10]. Nevertheless, a recent study demonstrates that cell intrinsic mechanisms are not the underlying mechanism of IL-1R1 host resistance in mice [11]. Although the precise mechanism is unclear, *in vivo*, a major function of IL-1R1-mediated signal seems to be to suppress necrotic tissue pathology in the lungs through complex interactions between both immune and non-immune stromal cells [2,12,13]. Importantly, the immunomodulatory function of IL-1β can be exploited for host-targeted therapeutic approaches [12].

The inflammasome complex consists of the specific Nucleotide binding and oligomerization domain-like receptor (NLR) or AIM2-like receptors (ALR), an adaptor protein ASC in many but not all inflammasomes and finally the protease caspase-1 (Casp1) which will get activated once the full inflammasome complex has formed. Casp1 will cleave the immature, pro-IL-1β to generate the truncated, mature IL-1β which is released by the cell [14–17]. Another potential outcome of the inflammasome activation is pyroptosis which is mediated by the cleavage of gasdermin D (GSDMD) by activated Casp1 or Casp11 [18–20]. The cleaved GSDMD will generate pores in the cell membrane which will lead to pyroptosis [21–23] (for review [24,25]).

Numerous NLRs and ALRs recognize cytosolic PAMPs or DAMPs but only NLRP3 mediates activation of the inflammasome in macrophages infected with Mtb [26–29]. The activation is dependent on phagocytosis of the bacteria, potassium efflux but not IFNR or P2X7R signaling nor presence of reactive oxygen species (ROS) or lysosomal rupture and release of cathepsins [29–31]. In contrast, a separate study implicated lysosomal rupture and cathepsin B in Mtb-mediated inflammasome activation [32]. The tyrosine kinase SYK is important for Mtb-mediated inflammasome activation but it is not known what its downstream target is [31].

The inflammasome activation mediated by Mtb is dependent upon the expression of the ESX-1 type-VII secretion system [28,30,33–35]. The ESX-1 secreted protein, EsxA, is capable of inducing the activation of the NLRP3 inflammasome [34] potentially via damaging the plasma membrane and subsequent potassium ion efflux [29]. The activation of the AIM2-inflammasome is inhibited by Mtb and most likely via introduction of effectors into the host cell cytosol, since the inhibition is dependent on ESX-1 [36]. This immunoevasion may have important consequences for virulence of Mtb since *Aim2*^-/-^ mice are more susceptible to Mtb infections [37]. The Mtb protein, Zmp1, was proposed to mediate inhibition of the inflammasome activation [9] but these findings could not be confirmed by another group [31]. In addition, the cell envelope-associated serine hydrolase, Hip1, inhibits the inflammasome activation by limiting TLR2-dependent cell signaling [38,39]. This inhibition is not specific for the inflammasome but includes many pro-inflammatory cytokines stimulated via TLR2-MyD88 signaling axis [38,39]. The *Hip1* Mtb mutant is attenuated in mice but, due to its pleiotropic effects, it is not certain if that is due to the inflammasome phenotype or to any of the other downstream targets of TLR-signaling [40].

Here, we further investigated the Mtb-mediated manipulation of the host cell inflammasome and discovered that Mtb can inhibit NLRP3-inflammasome activation via a mechanism that does not require its ESX-1 type VII secretion system. Furthermore, we demonstrate that the Mtb serine/threonine phosphokinase PknF is important for the inhibition of the NLRP3 inflammasome.

## Results

### Mtb inhibits the NLRP3 Inflammasome activation in an ESX-1-independent mechanism

**Fig 1:**
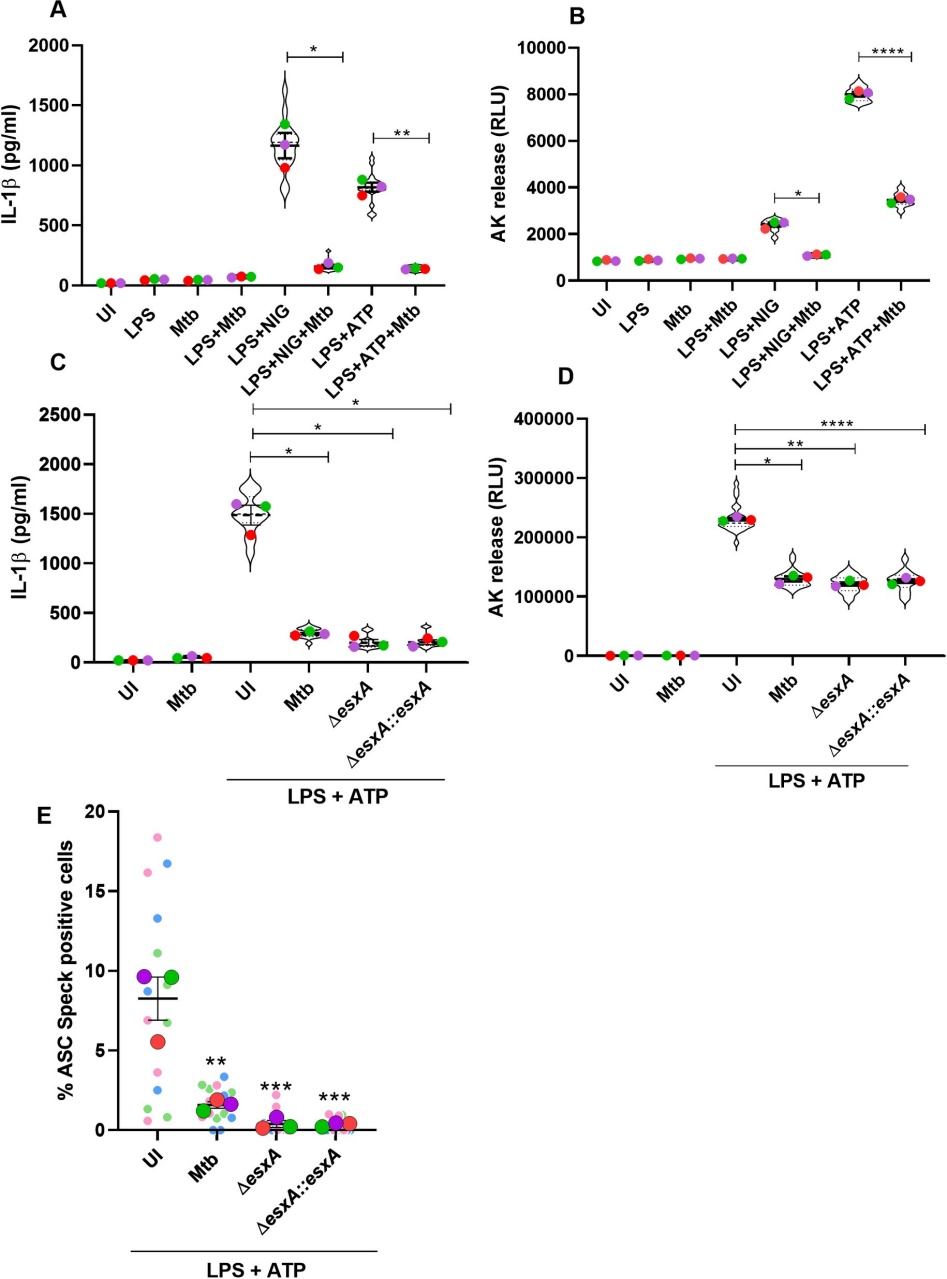
Mtb inhibits the NLRP3 Inflammasome activation in an ESX-1-independent mechanism. BMDMs were primed with 100ng/ml LPS for 4 h and concurrently infected with Mtb (CDC1551) at an MOI of 10 or left uninfected and then stimulated with two different NLRP3 inflammasome activators, Nigericin (NIG, 1μM) and ATP (0.1mM) for 30 min. Uninfected and untreated cells (UI), only LPS-treated (LPS), only Mtb-infected (Mtb) and LPS+Mtb-treated cells (LPS+Mtb) served as negative controls in the experiment. LPS+NIG and LPS+ATP are the positive controls for inducing NLRP3 inflammasome activation on uninfected cells. Cell supernatants were harvested after 30 min of stimulation and analyzed for (A) IL-1β release by ELISA (B) cell death by adenylate kinase (AK) release assay. BMDMs were primed with LPS (100ng/ml) for 4 h, or left unprimed, simultaneously infected with different Mtb (H37Rv) strains (Mtb, Δ*esxA*, and Δ*esxA*::*esxA***)** followed by treatment with the NLRP3 activator ATP (0.1mM, 30 min). Cell supernatants were harvested as before and analyzed for (C) IL-1β secretion and (D) cell death. Subsequently, cells were fixed, permeabilized and immunostained for ASC (Alexa Fluor 594, red), and (E) percentage of cells with ASC speck out of all cells were quantified. Data are representative of three independent biological replicates with n>4 in technical replicates per condition. Each colored data point represents average of technical replicates for each of the biological replicate (n = 3). Error bars represent mean ± SEM; *, p<0.05, **, p<0.01, ***, p<0.001, ****, p<0.0001.

LPS-primed bone marrow-derived macrophages (BMDMs) stimulated with different doses of NLRP3 agonists: Nigericin (40μM, 20μM, 10μM, 5μM, 1μM, 0.05 μM, 0.005 μM) or ATP (5mM, 2.5 mM, 1 mM, 0.1 mM, 0.01 mM, 0.001 mM) resulted in different levels of IL-1β secretion in a dose dependent manner. In order to study NLRP3 inflammasome inhibition by Mtb, 1μM Nigericin and 0.1mM ATP were selected because they are the lowest doses to induce a robust signal and thus might give us the best chance to observe inhibition (S1A and S1B Fig). LPS-primed BMDMs were infected with Mtb (CDC 1551) at a multiplicity of infection (MOI) of 10 for 4 h and subsequently stimulated with Nigericin (1μM) and ATP (0.1mM) for 30 min. Mtb infection resulted in a significant reduction in the amount of IL-1β secretion when compared to the uninfected, LPS-primed cells treated with either Nigericin or ATP (Fig 1A). LPS treatment alone, Mtb infection alone or LPS together with Mtb infection failed to produce any significant amount of IL-1β at this early timepoint (30 min postinfection). Cell death analysis by adenylate kinase (AK) release assay revealed that Mtb inhibits the cell death induced by NLRP3 inflammasome agonists (Fig 1B). Together, these results suggest that Mtb suppresses NLRP3 inflammasome activation and subsequent IL-1β secretion and cell death.

Next, we wanted to investigate whether the NLRP3 inflammasome inhibition is dependent on a functional ESX-1 secretion system. To address this question we performed infection with the following Mtb strains: Mtb, Δ*esxA*, and Δ*esxA*::*esxA*. Similarly to Mtb infection, both Δ*esxA*, and Δ*esxA*::*esxA* strains in the presence of NLRP3 inflammasome activator showed a significant reduction in IL-1β production along with cell death (Fig 1C and 1D). These data suggest that Mtb inhibits the NLRP3 inflammasome activation independently of the ESX-1 secretion system.

Furthermore, we performed detection of ASC speck formation by immunofluorescence microscopy as an additional read-out for inflammasome activation. Stimulation of LPS-primed uninfected BMDMs with ATP-induced formation of ASC specks (S2 Fig). However, stimulation of LPS-primed, Mtb, Δ*esxA*, and Δ*esxA*::*esxA* infected BMDMs with ATP showed a significant reduction in cells that formed ASC specks (S2 Fig). Quantitative analysis of the percentage of ASC specks positive cells from uninfected and Mtb-infected cells in the presence of NLRP3 inflammasome activators demonstrates that compared to uninfected and ATP-stimulated cells, Mtb infection leads to significant decrease in the number of ASC specks-positive cells which is independent of the ESX-1 secretion system (Fig 1E). These data suggest that Mtb inhibits the NLRP3 inflammasome activation by either directly preventing the formation of ASC specks or inhibiting an event upstream to NLRP3 inflammasome assembly.

### The deletion of *pknF* in Mtb leads to increased inflammasome activation and cell death in BMDM

We performed a gain-of-function genetic screen using *Nlrp3*^-/-^ host cells in order to specifically identify genes of Mtb that mediate the inhibition of the AIM2 inflammasome as we previously had discovered this capacity of Mtb [36]. One of the false positive hits we obtained during the screening was PknF which has no role in the inhibition of the AIM2 inflammasome but does mediate inhibition of the NLRP3 inflammasome as we will demonstrate here. PknF is one of the 11 eukaryotic-like serine/threonine protein kinases (STPK) encoded in the genome of Mtb [41]., We constructed a *pknF* deletion mutant in Mtb CDC1551 (*ΔpknF*) by specialized transduction [42] and confirmed the deletion of *pknF* by Southern hybridization (S3A Fig). Disruption of *pknF* showed no differences in *in vitro* growth rates of the bacterium when compared with the growth of the parental (Mtb) and *pknF* complemented strain (Δ*pknF*::*pknF*) in the Middlebrook 7H9 medium (S3B Fig). Thus, the data suggest that *pknF* gene deletion does not alter bacterial growth in standard culture conditions. Moreover, one-dimensional TLC analysis showed that, compared to wild-type Mtb and Δ*pknF*::*pknF* strain, deletion of *pknF* does not affect the PDIM levels, a cell wall associated surface lipid required for virulence (S3C Fig).

**Fig 2:**
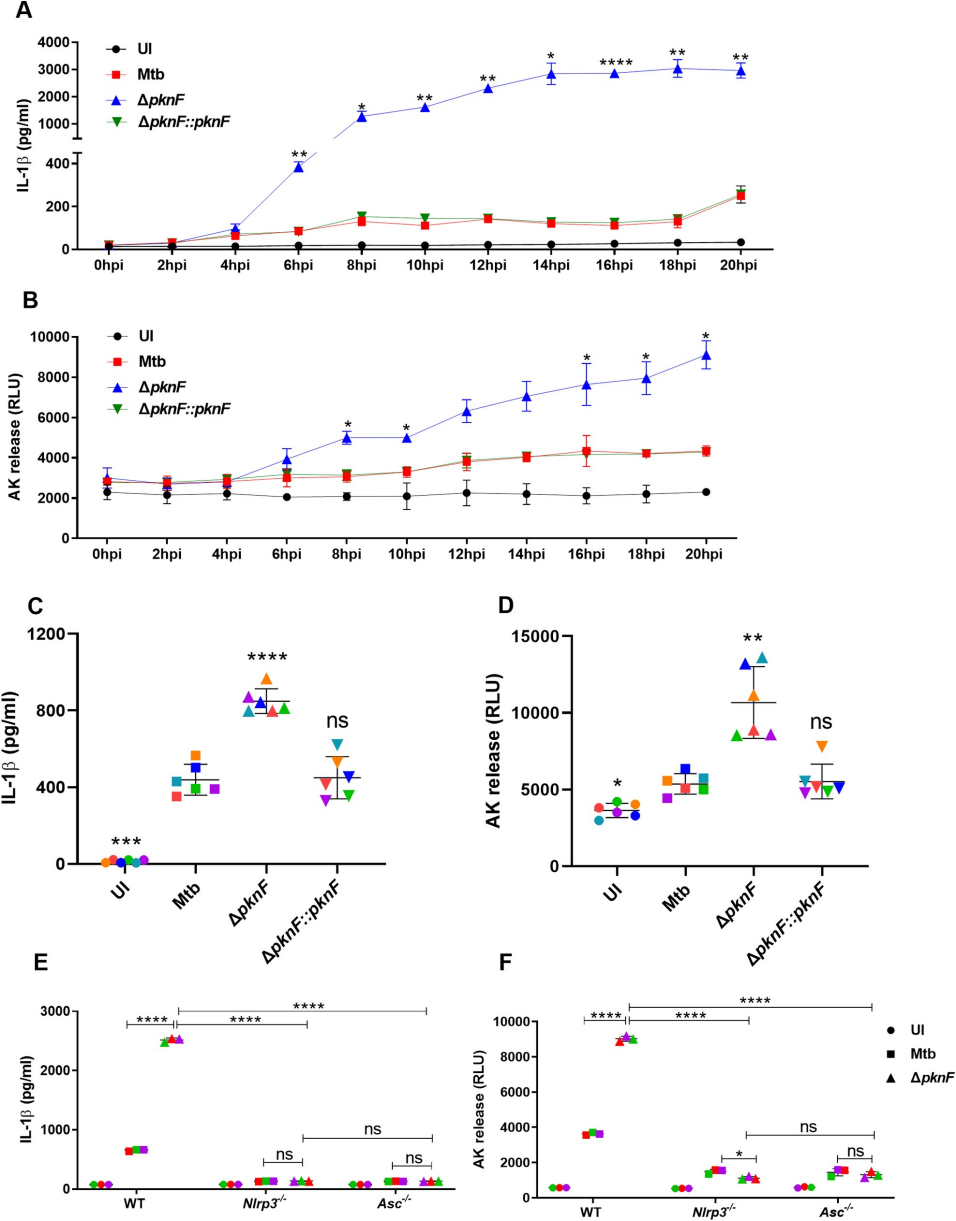
The deletion of *pknF* in Mtb leads to increased NLRP3 inflammasome and ASC dependent IL-1β production and cell death in BMDM. BMDMs were either left uninfected (UI) or infected with different Mtb (CDC1551) strains (Mtb, *ΔpknF* mutant and complement Δ*pknF*::*pknF*) at an MOI of 10 for 4h. For each timepoint cell supernatants were harvested from separate wells. (A) Secretion of IL-1β and (B) cell death was determined every 2 hpi until 20 hpi by ELISA and by quantification of the release of adenylate kinase (AK) respectively. hMDMs were either left uninfected (UI) or infected with different Mtb (CDC1551) strains (Mtb, *ΔpknF* mutant and complement Δ*pknF*::*pknF*) at an MOI of 10 for 4h. The culture supernatants were harvested at 20 hpi and analyzed for (C) IL-1β levels and (D) cell death by ELISA and AK release assay respectively. Data are representative of six independent biological replicates from two different donors. BMDMs derived from wild-type (WT), *Nlrp3*^-/-^, and, *Asc*^*-*/-^ mice were either left uninfected (UI) or infected as indicated with Mtb and *ΔpknF* mutant strain at an MOI of 10 for 4h. At 20 hpi (E) secreted IL-1β was measured by ELISA and (F) cell death was measured by AK release assay. Data are representative of three independent biological replicates. Error bars represent mean ± SD; *, p<0.05, **, p<0.01, ***, p<0.001, ****, p<0.0001, ns (non-significant).

Next, we performed a time course study on BMDMs infected with Mtb (CDC1551), the *ΔpknF* mutant and the complement Δ*pknF*::*pknF* strains at an MOI of 10 for 4 h. The cell free supernatants were collected every 2 hours post infection (hpi) for up to 20 hpi on separate wells. At 6 hpi was the earliest time point when the *ΔpknF* mutant, in contrast to Mtb and complement Δ*pknF*::*pknF*, showed a significant increase in the production of IL-1β. The analysis of ASC-speck formation at this timepoint confirmed the observance of speck-positive cells in *ΔpknF* mutant but not wild-type or complemented strains infected cells (S4B Fig). The levels of IL-1β increased until a plateau was reached at about 14 hpi (Fig 2A). Beside secretion of IL-1β, activation of inflammasome can trigger the release of another proinflammatory cytokine, IL-18 [17]. We found that BMDMs infected with the *ΔpknF* mutant showed a significant increase in the levels of IL-18 similar in magnitude and kinetic as demonstrated for IL-1β (S4A Fig). Correspondingly, cell death analysis revealed that the *ΔpknF* mutant, compared to Mtb and complement Δ*pknF*::pknF, induces a significant increase in cell death as early as 8 hpi that steadily rose over time until the end of the experiment at 20 hpi (Fig 2B). To determine whether existing or newly synthesized bacterial proteins lead to an increased IL-1β production after infection with the *ΔpknF* mutant, BMDMs were infected with heat killed (65°C or 80°C) Mtb and the *ΔpknF* mutant strains for 4 h. In contrast to live Mtb, the heat-killed *ΔpknF* mutant failed to induce IL-1β secretion and cell death (S5A and S5B Fig). Additionally, BMDMs were infected with chloramphenicol-treated or untreated bacteria, to ask whether newly synthesized Mtb proteins are required for the induction via the mutant. Secretion of IL-1β and cell death was significantly reduced from BMDMs infected with chloramphenicol-treated *ΔpknF* mutant (S5A and S5B Fig). Taken together these findings suggest that newly synthesized proteins of Mtb and the *ΔpknF* mutant are involved in either the initial activation of the inflammasome and/or the inhibition of the inflammasome activation. More detailed analyses are required to distinguish between these two interpretations.

In order to examine that *ΔpknF* mutant leads to increased inflammasome activation not only in mouse macrophages but also in human macrophages, human monocyte-derived macrophages (hMDMs) were infected with Mtb, the *ΔpknF* mutant and the complement Δ*pknF*::*pknF* strains at an MOI of 10 for 4 h. We used cells from 2 different donors and for each 3 independent experiments were performed (n = 6). The cell culture supernatants were harvested at 20 hpi and analyzed for IL-1β levels and cell death. Secretion of IL-1β and cell death was significantly increased from hMDMs infected with *ΔpknF* mutant when compared to Mtb and complement *ΔpknF*::*pknF*, (Fig 2C and 2D). These results suggest that *ΔpknF* mutant induces increase inflammasome activation in both mouse and human macrophages.

### The Mtb *pknF* mutant mediates NLRP3 inflammasome and ASC dependent IL-1β production and pyroptosis in BMDMs

Next, we investigated which inflammasome components are involved in increased production of IL-1β and cell death by the *ΔpknF* mutant. BMDMs derived from the wild-type, *Nlrp3*^-/-^, *Aim2*^*-/-*^, *Asc*^*-*/-^, *Ripk3*^-/-^and *Ripk3/Casp8*^*-/-*^ were infected with Mtb and *ΔpknF* mutant for 4 h and cell culture supernatants were harvested at 20 hpi and evaluated for IL-1β levels and cell death. Compared to IL-1β levels produced from wild-type, *ΔpknF* mutant infected BMDMs, the secretion of IL-1β was completely abolished when NLRP3 or ASC deficient BMDMs were infected with the *ΔpknF* mutant (Fig 2E). Conversely, compared to wild-type BMDMs infected with the *ΔpknF* mutant, IL-1β levels remained unaltered in *Aim2*^*-/-*^, *Ripk3*^*-/-*^ and *Ripk3/Casp8*^*-/-*^ macrophages (S6A and S6B Fig). Moreover, depletion of either NLRP3 or ASC showed a significant reduction in cell death in contrast to *ΔpknF* mutant-infected wild-type BMDMs (Fig 2F). On the other hand, cell death remained unaffected in *Aim2*^*-/-*^, *Ripk3*^*-/-*^and *Ripk3/Casp8*^*-/-*^ macrophages (S6C and S6D Fig). Altogether, the results indicate that NLRP3 and ASC are required for enhanced production of IL-1β and increased cell death during *ΔpknF* mutant infection.

### The Mtb *pknF* mutant is deficient in inhibiting NLRP3 inflammasome activation

**Fig 3:**
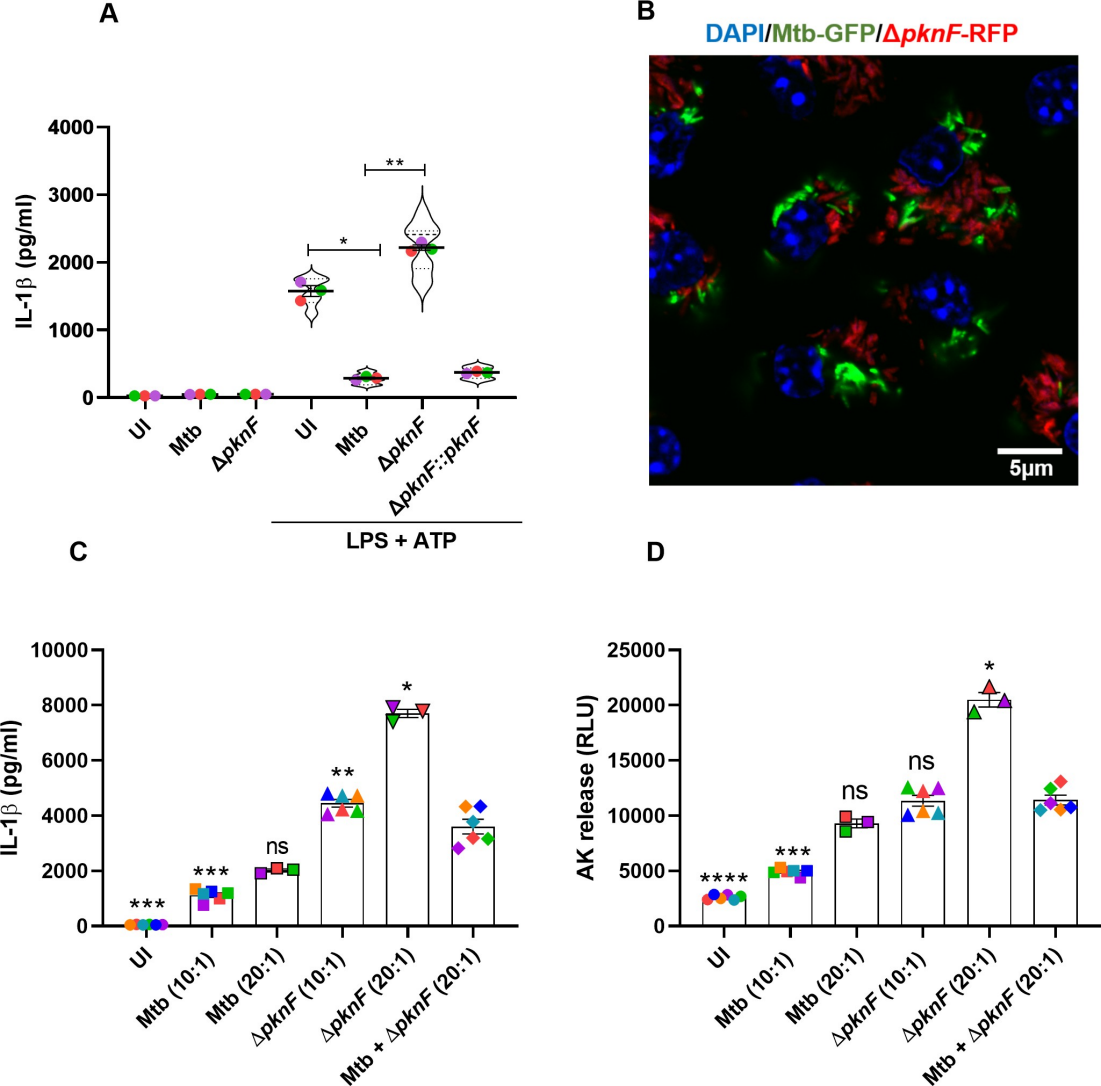
The Mtb-mediated inhibition of the NLRP3 inflammasome activation in BMDMs is dependent upon PknF. BMDMs were either left unprimed or primed with LPS (100ng/ml, 4 h), concomitantly infected where needed with different Mtb (CDC1551) strains (Mtb, *ΔpknF* mutant and complement Δ*pknF*::*pknF*), prior to treatment with ATP (0.1mM, 30 min) to activate the NLRP3 inflammasome. Unprimed/uninfected (UI) cells, Mtb and *ΔpknF* mutant infection alone served as control in the experiment. LPS-primed UI cells stimulated with ATP were used as positive control. Cell supernatants were harvested after 30 min of stimulation and analyzed for (A) IL-1β release by ELISA. Data are representative of three independent biological replicates with n = 6 in technical replicates per condition. Each colored data point represents average of technical replicates for each of the biological replicate (n = 3). BMDMs were either left uninfected (UI) or mono-infected at two different MOI (10:1 and 20:1) or co-infected at a MOI of 10:1 for each strain for 4 h with Mtb-*gfp* and Δ*pknF-rfp* mutant CDC1551 strains. After 4 h infection, Co-infected cells were observed by (B) fluorescence microscopy. Scale bar 5μm. The cell culture supernatants were harvested at 20 hpi and analyzed for (C) IL-1β levels and (D) cell death by ELISA and AK release assay respectively. Data are representative of six independent biological replicates. Error bars represent mean ± SEM; *, p<0.05, **, p<0.01***, p<0.001, ****, p<0.0001, ns (non-significant).

In order to test if PknF is actually involved in the inhibition of NLRP3 inflammasome activation in BMDMs, we infected untreated and LPS-treated BMDMs with Mtb, the *ΔpknF* mutant and complemented strains for 4 h and subsequently stimulated the cells with ATP (0.1mM) for 30 min to activate the NLRP3 inflammasome. The cell culture supernatants were harvested and analyzed for IL-1β levels. We found that in contrast to Mtb and the complemented *ΔpknF*::*pknF* strains, he *ΔpknF* mutant strain fails to inhibit the secretion of IL-1β induced by ATP (Fig 3A). At this early time point post infection (30 min), neither Mtb nor the *ΔpknF* mutant alone induced any significant secretion of IL-1β (Fig 3A) These results suggest that in mouse macrophages, PknF plays an important role in inhibiting the activation of the NLRP3 inflammasome.

Next, we investigated when co-infecting BMDMs with Mtb and the *ΔpknF* mutant, if Mtb is able to inhibit the secretion of IL-1β and cell death induced by the *ΔpknF* mutant. Thus, to test whether Mtb inhibits the activation of the NLRP3 inflammasome induced by *ΔpknF* mutant, we mono-infected BMDMs at two different MOI (10:1 and 20:1) and co-infected BMDMs at a MOI of 10:1 for each strain for 4 h with Mtb-*gfp* and Δ*pknF-rfp* mutant strains. Co-infection was established by fluorescence microscopy (Fig 3B). We found that secretion of IL-1β from mono-infected cells at a MOI 20:1 (Mtb 2017 ± 52.63 pg/ml and *ΔpknF* mutant 7703 ± 153.4 pg/ml) showed additive effect when compared to MOI 10:1 (Mtb 1121 ± 85.23 pg/ml and *ΔpknF* mutant 4452 ± 137 pg/ml). Similarly, cell death in mono-infected cells when compared to MOI 10:1 (Mtb 4919 ± 124.8 RLU and *ΔpknF* mutant 11351 ± 492.3 RLU) showed additive effect at a MOI 20:1 (Mtb 9305 ± 388.9 RLU and 20504 ± 652.7 RLU). Hence the co-infected cells at MOI 10:1 for each strain should show a predicted value that will be the sum of Mtb and *ΔpknF* mutant infection at MOI 10:1 if there is independence between the two infections. Our data show that when compared to the Mtb + *ΔpknF* predicted values of 5573 ± 210.3 pg/ml and 16270 ± 418.6 RLU for IL-1β and cell death, respectively, the actual values of the Mtb + *ΔpknF* co-infected cells showed a significant reduction in the release of IL-1β (3604 ± 263.2 pg/ml; p value < 0.0001) and cell death (11423 ± 446 RLU; p value < 0.0001) (Fig 3C and 3D). This data suggests that there is antagonism between Mtb and the *ΔpknF* mutant within the co-infected cell in regard to activation of the NLRP3 inflammasome.

### The impact of Casp1, Casp11 and GSDMD on the IL-1β secretion and pyroptosis induced by the Mtb *pknF* mutant

**Fig 4:**
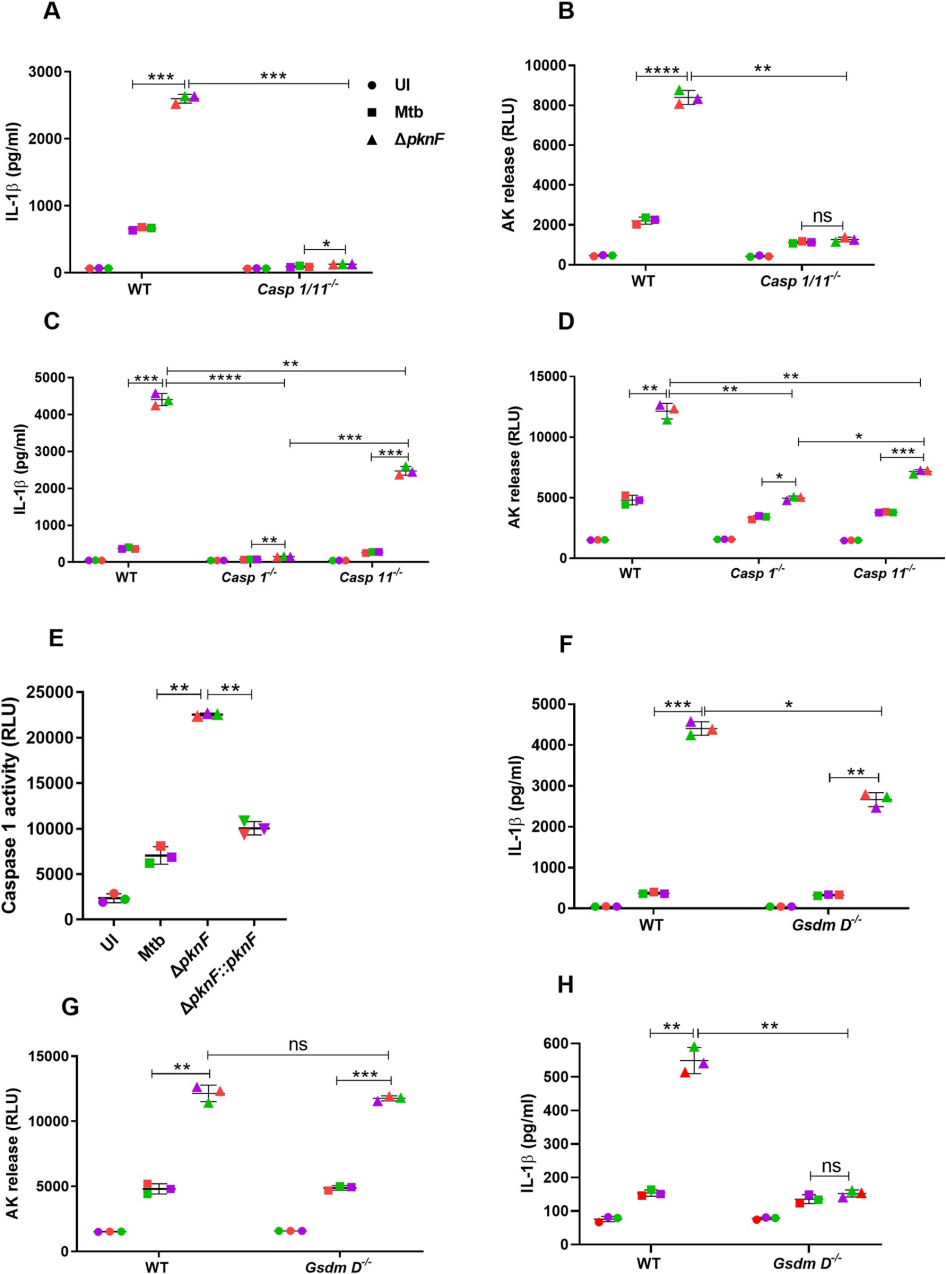
The impact of Casp1, Casp11 and GSDMD on the increased production of IL-1β and cell death by the *pknF* Mtb mutant. BMDMs derived from wild type (WT), *Casp1*^-/-^, *Casp11*^-/-^, *Casp1*^-/-^*/11*^*-/-*^ and *GSDMD*^*-/-*^ mice were either left uninfected (UI, solid circles) or infected with CDC1551 Mtb wild-type (solid squares) and *ΔpknF* mutant (solid upright triangles) at an MOI of 10 for 4h. The cell free supernatants were assessed for IL-1β secretion by ELISA at 20 hpi (A, C, F) and at 6 hpi (H) and (B, D, G) cell death by measuring the release of adenylate kinase (AK). WT BMDMs were either left uninfected (UI, solid circles) or infected with different Mtb (CDC1551) strains (Mtb, *ΔpknF* mutant and complement Δ*pknF*::*pknF* [solid inverted triangles]) and supernatants collected at 20 hpi were analyzed for (E) caspase-1 activity by caspase-1-Glo assay. Data are representative of three independent biological replicates. Each colored data point represents average of technical replicates for each of the biological replicate (n = 3). Error bars represent mean ± SD; *, p<0.05, **, p<0.01, ***, p<0.001, ****, p<0.0001, ns (non-significant).

The assembly of the NLRP3 inflammasome complex in mouse macrophages leads to activation of inflammatory caspases, include Casp1 (canonical pathway) and Casp11 (non-canonical pathway) [17]. Active Casp1 and Casp11 drive pyroptosis through the cleavage of gasdermin D (GSDMD) into two fragments, the N-terminal pore-forming domain and a C-terminal repressor domain [24,25]. We infected the wild-type, *Casp1*^-/-^, *Casp11*^-/-^, *Casp1/11*^*-/-*^ and *GsdmD*^*-/-*^ BMDMs with Mtb and *ΔpknF* mutant for 4 h and cell culture supernatants were harvested at 20 hpi and assessed for IL-1β levels and cell death. *Casp1/11* double knockout BMDMs failed to secrete IL-1β and undergo cell death during *ΔpknF* mutant infection in contrast to infection of wild-type BMDMs with *ΔpknF* mutant (Fig 4A and 4B). To distinguish between canonical and non-canonical inflammasome activation targeted by the *ΔpknF* mutant, IL-1β levels were measured in BMDMs deficient in either *Casp1* or *Casp11*. Infection of wild-type BMDMs with *ΔpknF* mutant induced a significant production of IL-1β, whereas secretion of IL-1β was completely abrogated in *Casp1*^-/-^ BMDMs while only partially impaired in *Casp11*^-/-^ BMDMs (Fig 4C). Moreover, cell death compared to wild-type BMDMs was significantly reduced in both *Casp1*^-/-^ and *Casp11*^-/-^ BMDMs during infection with *ΔpknF* mutant (Fig 4D). Additionally, caspase-1 activity assay revealed that *ΔpknF* mutant results in increased activation of caspase-1 in BMDMs, in contrast to infection with Mtb and complement *ΔpknF*::*pknF* (Fig 4E). Taken together, these results suggest that primarily Casp1 with a minor contribution of Casp11 are required for the increased maturation and release of IL-1β in *ΔpknF* mutant infected BMDMs.

Increases in phagosome damage may lead to Casp11 activation and hence we wanted to analyze the phagosomal integrity in cells infected with the different Mtb strains. BMDMs were infected with Mtb and the *ΔpknF* mutant strains at an MOI of 10 for 4 h. At 6hpi the recruitment of TAX1BP1, an autophagy adapter and a galectin-8 interacting protein, to Mtb/*ΔpknF* mutant was examined by immunofluorescence microscopy. Colocalization analysis performed using the JaCoP plugin for Image J revealed that there was no significant difference in the recruitment of TAX1BP1 during the Mtb and the *ΔpknF* mutant infection (S7A and S7B Fig). Because we observed comparable levels of colocalization, suggesting that it is unlikely that phagosomal membrane rupture and/or cytosolic access is required for the increased inflammasome activation during *ΔpknF* mutant infection. Furthermore, we also performed another assay to investigate the possibility of increased cytosolic access during *ΔpknF* mutant infection. BMDMs derived from the wild-type and *Asc*^*-*/-^ mice were infected with Mtb and the *ΔpknF* mutant strains at an MOI of 10 for 4 h and cell culture supernatants were harvested at 20 hpi and analyzed for IFN-β levels. Compared to wild-type BMDMs infected with the *ΔpknF* mutant, the difference in IFN-β levels between Mtb and the mutant was completely abolished in ASC-deficient BMDMs (S7C Fig). These results indicate that the increase in IFN-β levels observed in wild-type BMDMs during *ΔpknF* mutant infection is solely due to increase in cell death induced by this mutant; because, in BMDMs deficient in ASC the *ΔpknF* mutant showed no increase in cell death compared to Mtb and then also no significant increase in production of IFN-β when compared to Mtb (S7C Fig). Together, these data suggest that when compared to Mtb, the *ΔpknF* mutant does not induce increased phagosomal membrane rupture and/or cytosolic access.

Next, we demonstrated a role for the pore-forming substrate of Casp1 and -11, GSDMD, since *GsdmD*^*-/-*^ BMDMs demonstrated a partial reduction in IL-1β production following *ΔpknF* mutant infection after 20 hpi when compared to wild-type BMDMs (Fig 4F) but no differences were observed for cell death (Fig 4G). The early secretion of IL-1β is fully dependent upon GSDMD but later secretion of IL-1β follows a GSDMD-independent pathway [43]. We repeated the experiment and harvested the supernatants at an earlier timepoint (6 hpi) in order to capture the GSDMD-dependent secretion of IL-1β. We showed that the secretion of IL-1β was abolished at 6 hpi in *GsdmD*^*-/-*^ BMDMs, whereas the mutant induced significant increase in IL-1β secretion in wild-type BMDMs (Fig 4H). Collectively, these results suggest that induction of cell death by the *ΔpknF* mutant was independent of GSDMD and *ΔpknF* mutant infection results in both GSDMD-dependent and -independent secretion of IL-1β. In the absence of GSDMD, Casp1 can induce an alternative cell death pathway that involves Casp3 which could explain our observed lack of phenotype of the GSDMD-deficient cells [44]. BMDMs derived from the wild-type and *GsdmD*^*-/-*^ were infected with Mtb and *ΔpknF* mutant in the presence or absence of Casp3 inhibitor Z-DEVD-FMK. Compared to untreated cells, cells pre-treated with Casp3 inhibitor showed significant reduction in the cell death in both WT and *GsdmD*^*-/-*^ BMDM during infection with either Mtb or *ΔpknF* mutant (S8A Fig). Compared to untreated cells, cells pre-treated with Casp3 inhibitor showed significant reduction in the secretion of IL-1β in WT BMDM, however there was no significant difference in IL-1β release in *GsdmD*^*-/-*^ BMDM during *ΔpknF* mutant infection (S8B Fig). In addition to Casp3 inhibitor studies, we also performed analysis of cell death and IL-1β secretion from WT and *Casp3*^*-/-*^ BMDMs infected with Mtb and *ΔpknF* mutant strains. Compared to wild-type BMDMs infected with the *ΔpknF* mutant, cell death and IL-1β levels were significantly decreased in *Casp3*^*-/-*^ macrophages (S8C and S8D Fig). These results suggest that Casp3 is partially involved in inducing cell death and IL-1β secretion in BMDM during *ΔpknF* mutant infection.

### The Mtb *pknF* mutant induces increased activation of the NLRP3 inflammasome that is independent of the priming step

**Fig 5:**
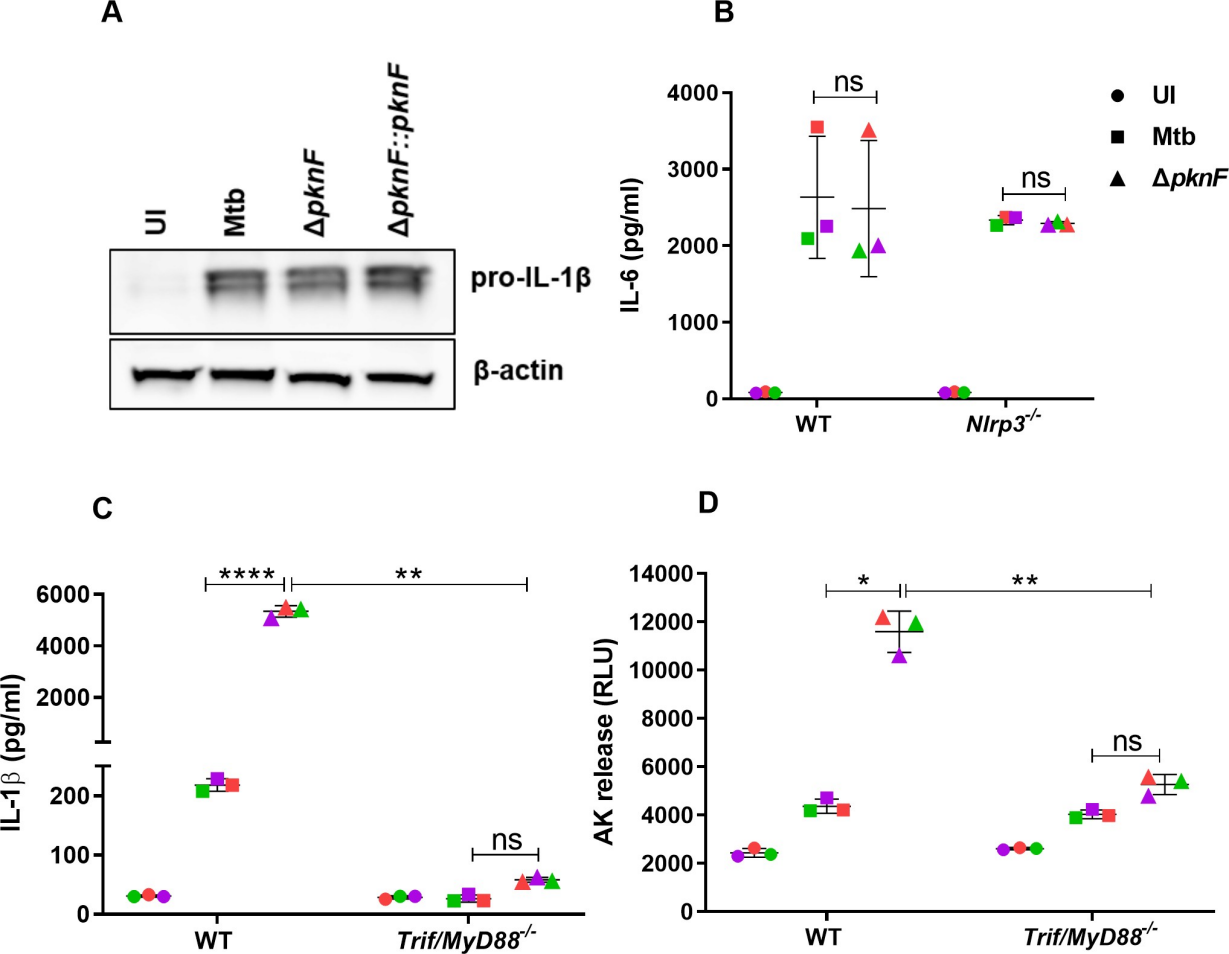
The *pknF* Mtb mutant induces increased NLRP3 inflammasome activation independently of transcriptional priming. BMDMs were either left uninfected (UI) or infected with different CDC1551 Mtb strains (Mtb, *ΔpknF* mutant and complement Δ*pknF*::*pknF*) at an MOI of 10. Cell lysates were prepared at 4 hpi and performed (A) Western blot analysis for pro-IL-1β (34 kDa) and β-actin (42 kDa). Data is representative of one independent experiment out of three independent biological replicates. BMDMs derived from wild type (WT), *Nlrp3*^-/-^ and *Trif*^-/-^*/Myd88*^-/-^ mice were either left uninfected (UI, solid circles) or infected with Mtb (solid squares) and *ΔpknF* mutant (solid upright triangles). Culture supernatants were harvested at 20 hpi and analyzed for (B) IL-6 secretion by ELISA (C) IL-1β secretion and (D) cell death by measuring the release of cytosolic enzyme, adenylate kinase (AK). Data are representative of three independent biological replicates. Each colored data point represents average of technical replicates for each of the biological replicate (n = 3). Error bars represent mean ± SD; *, p<0.05, **, p<0.01, ****, p<0.0001, ns (non-significant).

Next, we assessed if the increase in inflammasome activation by the *ΔpknF* mutant is due to an increase in activation of the priming step when compared to Mtb-infected cells. BMDM cell lysates were collected at 4 hpi and subjected to Western blot analysis to compare the expression levels of pro-IL-1β during infection with Mtb, the *ΔpknF* mutant and complement Δ*pknF*::*pknF* strains (all CDC1551 background). At 4 hpi, there was no significant difference in the pro- IL-1β expression levels among all the three strains (Fig 5A). Furthermore, IL-6 production was not significantly different in both wild-type and *Nlrp3*^-/-^ BMDMs following *ΔpknF* mutant infection when compared to wild-type Mtb or complemented mutant strains (Fig 5B). These results strongly suggest that the observed increase of IL-1β production and pyroptosis during infection with *ΔpknF* mutant is due to an increase in inflammasome activation (signal 2) but not due to an increase in pro-inflammatory signaling (signal 1). Previous studies have implicated the role of adaptor Myd88 and TRIF in transcriptional and post-transcriptional regulation of the priming step [45–47]. To examine the role of Myd88 and TRIF during *ΔpknF* mutant infection, *Myd88/TRIF* double KO BMDMs were infected with Mtb and *ΔpknF* mutant for 4 h and supernatants were harvested at 20 hpi and assessed for IL-1β levels and cell death. The differences in IL-1β levels and cell death between wild-type Mtb and the *ΔpknF* mutant were abolished in TRIF/Myd88^-/-^ BMDMs following *ΔpknF* mutant infection (Fig 5C and 5D).

### NLRP3 inflammasome activation induced by the *pknF* Mtb mutant is dependent on potassium and chloride efflux but independent of calcium influx

**Fig 6:**
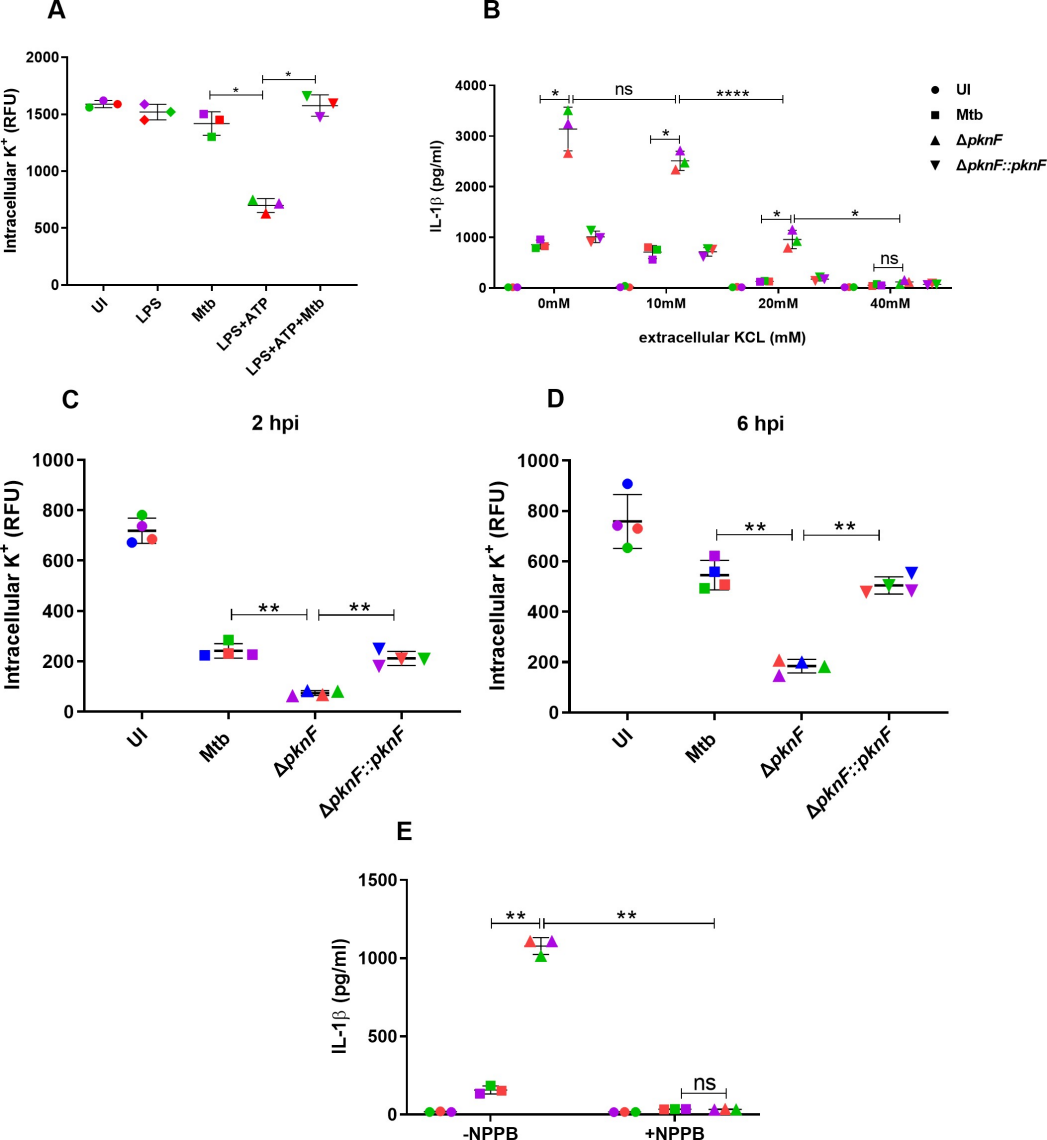
Potassium and chloride efflux are essential triggers for NLRP3 inflammasome activation in *pknF* Mtb mutant infected BMDMs. BMDMs were primed with LPS (100ng/ml, 4 h) followed by stimulation with NLRP3 activator, ATP (0.1mM, 30 min), when indicated, cells were infected with CDC1551 Mtb before stimulation with ATP. Uninfected cells, LPS treatment alone and Mtb infection alone were used as controls in the experiment. After 30 min of ATP stimulation (A) intracellular levels of K^+^ was measured by the FluxOR II green potassium ion channel assay. BMDMs were either left untreated (0 mM) or treated with increasing concentrations of extracellular KCL (10 mM, 20 mM and 40 mM) and then infected with different CDC1551 Mtb strains (Mtb, *ΔpknF* mutant and complement Δ*pknF*::*pknF*). Culture supernatants were harvested at 20 hpi and analyzed for (B) IL-1β secretion by ELISA. BMDMs were either left uninfected (UI) or infected with different Mtb strains (Mtb, *ΔpknF* mutant and complement Δ*pknF*::*pknF*) at an MOI of 10 for 4h and intracellular K^+^ concentration was measured at (C) 2 hpi and (D) 6 hpi by FluxOR II green potassium ion channel assay. BMDMs were either left untreated (-NPPB) or treated with chloride channel blocker, NPPB (100μM) and then infected with Mtb (solid squares) and *ΔpknF* mutant (solid upright triangles). Culture supernatants were harvested at 20 hpi and analyzed for (E) IL-1β secretion by ELISA. Data are representative of three independent biological replicates. Each colored data point represents average of technical replicates for each of the biological replicate (n = 3). Error bars represent mean ± SD; *, p<0.05, **, p<0.01, ****, p<0.0001, ns (non-significant).

Previous studies have described an important role for ion fluxes, including K^+^ efflux, Cl^-^ efflux and Ca^2+^ influx in activating the NLRP3 inflammasome [17]. We addressed the role of K^+^ efflux in Mtb-mediated NLRP3 inflammasome inhibition by determining the levels of intracellular K^+^ in Mtb-infected cells challenged with ATP. Stimulation of LPS-treated BMDMs with ATP showed a significant reduction in intracellular K^+^ concentration suggesting increased K^+^ efflux, whereas the Mtb infection prevented ATP-induced decrease in intracellular K^+^ concentration. These results thus demonstrate that Mtb inhibits the NLRP3 inflammasome activation by preventing either directly or indirectly the K^+^ efflux (Fig 6A). To investigate whether any of these ion fluxes are involved in the *ΔpknF* mutant-induced NLRP3 inflammasome activation, BMDMs were infected with Mtb and *ΔpknF* mutant in the presence or absence of defined inhibitors followed by analysis of IL-1β levels. Treatment of *ΔpknF* mutant-infected BMDMs with increasing concentration of extracellular KCL resulted in significant reduction in the secretion of IL-1β and high concentration of extracellular K^+^ (40mM) completely abolished the IL-1β production (Fig 6B). Moreover, we measured the intracellular K^+^ concentration at 2 hpi and 6 hpi in Mtb, *ΔpknF* mutant and complement Δ*pknF*::*pknF* infected BMDMs. The *ΔpknF* mutant, in contrast to Mtb and complement Δ*pknF*::*pknF* strains, induced a significant decrease in levels of intracellular K^+^ at both 2 hpi and 6 hpi (Fig 6C and 6D). These results demonstrate that K^+^ efflux is required for NLRP3 inflammasome activation in *ΔpknF* mutant infected BMDMs which is not surprising since most NRLP3 inflammasome activation pathways involve K^+^ efflux. In the presence of the chloride channel blockers, NPPB and DIDS, the *ΔpknF* mutant showed significant decrease in IL-1β production (Figs 6E and S9A). These data suggest that in addition to K^+^ efflux, Cl^-^ efflux is also an essential trigger for NLRP3 inflammasome activation in *ΔpknF* mutant infected BMDMs. To determine the role of Ca^2+^ signaling in *ΔpknF* mutant-induced NLRP3 inflammasome activation, BMDMs were treated with BAPTA-AM, a strong Ca^2+^ chelator and supernatants were examined for IL-1β levels. BAPTA-AM failed to inhibit the production of IL-1β in BMDMs during *ΔpknF* mutant infection (S9B Fig). Furthermore, in comparison to BMDMs stimulated with ATP that revealed a significant increase in the levels of intracellular Ca^2+^, BMDMs infected with Mtb, the *ΔpknF* mutant and complement Δ*pknF*::*pknF* showed no significant difference in the mobilization of intracellular Ca^2+^ at 4 hpi or at 6 hpi (S9C and S9D Fig). These results indicate that the *ΔpknF* mutant-induced NLRP3 inflammasome activation in BMDMs is independent of Ca^2+^ influx.

### Reactive oxygen species regulate the NLRP3 inflammasome activation in *pknF* Mtb mutant-infected BMDMs

**Fig 7:**
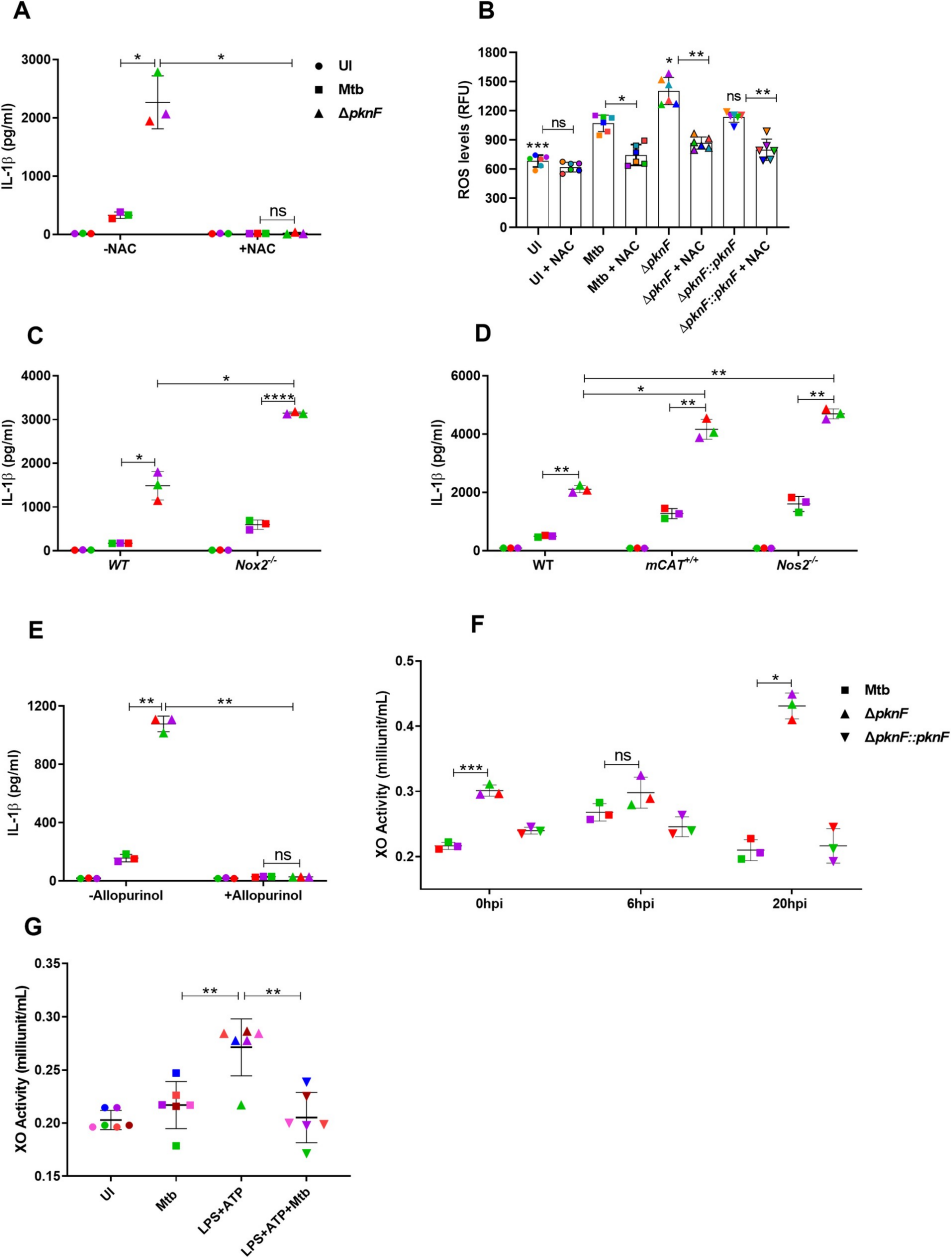
NLRP3 inflammasome activation by *pknF* Mtb mutant is dependent on xanthine oxidase (XO)-mediated generation of reactive oxygen species (ROS). BMDMs were either left untreated (-NAC) or treated with ROS scavanger, NAC (10mM) and then infected with CDC1551 Mtb wild-type and *ΔpknF* mutant. Culture supernatants were harvested at 20 hpi and analyzed for (A) IL-1β secretion by ELISA. BMDMs were either left untreated (-NAC) or treated with ROS scavanger, NAC (10mM) and then infected with different CDC1551 Mtb strains (Mtb, *ΔpknF* mutant and complement Δ*pknF*::*pknF*). At 6hpi cells were measured for (B) intracellular level of ROS by CellROX Green reagent. Data are representative of six independent biological replicates. BMDMs derived from wild type (WT), *Nox2*^-/-^, *Nos2*^*-/-*^ and *mCAT*^*+*/+^ mice were either left uninfected (UI, solid circles) or infected with Mtb (solid squares) and *ΔpknF* mutant (solid upright triangles) at an MOI of 10 for 4h. Culture supernatants were harvested at 20 hpi and analyzed for (C, D) IL-1β secretion by ELISA. BMDMs were either left untreated (-Allopurinol) or treated with XO inhibitor, allopurinol (250μg/ml) and then infected with Mtb and *ΔpknF* mutant. Culture supernatants were harvested at 20 hpi and analyzed for (E) IL-1β secretion by ELISA. BMDMs were infected with different Mtb strains (Mtb, *ΔpknF* mutant and complement Δ*pknF*::*pknF*) at an MOI of 10 for 4h and cell lysates prepared at 0 hpi, 6 hpi and 20 hpi were measured for (F) XO enzyme activity. BMDMs were primed with LPS (100ng/ml, 4 h) followed by treatment with NLRP3 activator, ATP (0.1mM, 30 min), when required, cells were infected with Mtb before stimulation with ATP. Uninfected cells and Mtb infection alone were used as controls in the experiment. After 30 min of ATP stimulation, cell lysates were assessed for (G) XO enzyme activity. Data are representative of three independent biological replicates. Each colored data point represents average of technical replicates for each of the biological replicate (n = 3). Error bars represent mean ± SD; *, p<0.05, **, p<0.01, ***, p<0.001, ****, p<0.0001, ns (non-significant).

The activation of the NLRP3 inflammasome is induced in response to increased intracellular generation of reactive oxygen species (ROS) and lysosomal destabilization [17]. To explore the role of ROS in *ΔpknF* mutant-mediated NLRP3 inflammasome activation, BMDMs were infected with Mtb and *ΔpknF* mutant in the presence or absence of ROS scavengers followed by evaluation of IL-1β levels. *ΔpknF* mutant*-*induced IL-1β secretion from BMDMs was abolished and significantly diminished upon treatment with both N-acetyl cysteine (NAC) (Fig 7A) and Tempol (S10A Fig) respectively. Moreover intracellular ROS levels were quantified at 6hpi in BMDMs infected with Mtb, the *ΔpknF* mutant and complement Δ*pknF*::*pknF*. *ΔpknF* mutant*-*induced significant increase in the amounts of ROS at 6 hpi when compared to Mtb and complement *ΔpknF*::*pknF*, (Fig 7B). NAC was added to both uninfected and infected BMDMs. NAC treatment inhibited ROS generation triggered by the *ΔpknF* mutant, confirming that the increased production of ROS was induced by the *ΔpknF* mutant (Fig 7B). These results indicate that generation of ROS is crucial in activation of NLRP3 inflammasome during *ΔpknF* mutant infection.

Recent studies demonstrated that there are various intracellular sources for ROS generation: 1) NADPH oxidase complex, 2) mitochondrial ROS and 3) xanthine oxidase (XO) [48]. To identify the major source of ROS in *ΔpknF* mutant-infected BMDMs, BMDMs derived from the wild-type, *Nox2*^-/-^, *Nos2*^*-/-*^, and *mCAT*^*+*/+^, were infected with Mtb and *ΔpknF* mutant followed by analysis of IL-1β in culture supernatants. In contrast to wild-type BMDMs, IL-1β secretion was significantly up regulated in *Nox2*^-/-^, *Nos2*^*-/-*^, and *mCAT*^*+*/+^ BMDMs during *ΔpknF* mutant infection (Fig 7C and 7D). These results indicate that neither ROS generated by the NADPH oxidase complex nor the mitochondria contribute to *ΔpknF* mutant mediated NLRP3 inflammasome activation. The increase in inflammasome activation in *Nox2*^-/-^ cells after infection with Mtb has been previously reported [49].

We next investigated the role of XO during *ΔpknF* mutant infection. Allopurinol, a selective inhibitor for XO, was used to block the XO activity. Allopurinol inhibits the IL-1β secretion in BMDMs induced by LPS/Nigericin and LPS/ATP (S10B Fig). We found that XO inhibition also abolished *ΔpknF* mutant induced release of IL-1β into culture supernatants (Fig 7E). In addition to inhibitor studies, we also measured the XO activity in Mtb, *ΔpknF* mutant and complement Δ*pknF*::*pknF* infected cell lysates at 0 hpi, 6 hpi and 20 hpi. *ΔpknF* mutant in comparison to Mtb and complement Δ*pknF*::*pknF* induced significant increase in the XO activity at 0 hpi and 20 hpi, however there was no significant difference at 6 hpi (Fig 7F). Taken together, these results demonstrate that during infection with the *ΔpknF* mutant, XO plays an important role in the generation of ROS that in turn is responsible for activation of NLRP3 inflammasome and increased production of IL-1β. We also addressed the role of XO in Mtb-mediated NLRP3 inflammasome inhibition by measuring the XO activity in Mtb-infected cells stimulated with ATP. ATP-stimulated BMDMs showed a significant increase in XO activity suggesting increased ROS production, however Mtb infection prevented ATP-induced increase in XO activity and as a result inhibited ROS production (Fig 7G). These results suggest that Mtb inhibits activation of NLRP3 Inflammasome by directly repressing XO activity or an event upstream of the activation of XO activity. Finally, to address the role of lysosomal cathepsin B in regulating the activation of NLRP3 inflammasome during *ΔpknF* mutant infection, release of cathepsin B was measured at 0, 2, 4, 6, 20 and 24 hpi. *ΔpknF* mutant showed increased intracellular cathepsin B activity at 24 hpi, though there were no significant differences in the cathepsin B activity at 0, 2, 4, 6, and 20 hpi (S10C Fig). These results reveal that lysosomal cathepsin B release is not essential for *ΔpknF* mutant induced NLRP3 inflammasome activation.

## Discussion

IL-1β is important for the successful host response to Mtb infections as demonstrated by the increased susceptibility of *Il1r1*^-/-^ and *Il1b*^-/-^ mice to Mtb [3,4,6,26]. The finding that *Asc*^-/-^ and *Casp1/11*^-/-^ mice are significantly more resistant than *Il1b*^-/-^ and produce similar amounts of IL-1β when compared to wild-type mice suggests that inflammasome-independent IL-1β production is the main driver of host protection to Mtb [26,27]. This could be interpreted as strong evidence that the host cell inflammasome having no, or only a minor, function in host defense. An alternative interpretation would be that Mtb is very proficient in inhibiting the inflammasome activation and that thus abolishing inflammasome function in mice does not further increase the virulence of Mtb. In support of this hypothesis Mtb was shown to mask its cell surface to avoid recognition by host cell TLRs which can, among other things, increase expression of pro-IL-1β, NLRP3 and AIM2 and thus provide the signal 1 for inflammasome activation [50]. In addition, Mtb inhibits activation of the AIM2-inflammasome via a mechanism that requires its ESX-1 secretion system [36]. In the current study, we now demonstrate that Mtb is able to potently oppose NLRP3-inflammasome activation via external stimuli (LPS+ATP/Nigericin) (Fig 1A and 1B). This is somewhat surprising since previous reports showed that the NLRP3-inflammasome is the main driver of inflammasome-mediated IL-1β production in macrophages and dendritic cells infected with Mtb *ex vivo* [27–29]. The induction of Mtb-mediated NLRP3-inflammasome activation is dependent upon ESX-1 expression [27–29]. The ESX-1 system is crucial for the permeabilization of the phagosomal membrane and for the access of Mtb effectors into the host cell [51]. It is thus likely that the NLRP3-inflammasome detects cell stress associated with Mtb-mediated host cell manipulation and therefore presents a logical target for immune evasion and suppression by Mtb.

Importantly, we identify PknF, a serine/threonine phosphokinase, as a Mtb component mediating NLRP3 inflammasome inhibition. First of all, it was important to demonstrate that PknF is actually needed for inhibition of the inflammasome as opposed to the *PknF* Mtb mutant just inducing more NLRP3-inflammasome activation when compared to wild-type Mtb (Fig 3A). Next, we showed that, different from the Mtb *Hip1* mutant, the *PknF* deletion does not result in increased activation of signal 1, since neither IL-6 secretion nor pro-IL-1β protein levels are changed in the mutant compared to wild-type Mtb (Fig 5A and 5B). The *Hip1* mutant is attenuated in mice [40] but the reason for this attenuation could be due to increase inflammasome activation and/or increased pro-inflammatory cytokine signaling (e.g. TNF). Our identification of an Mtb mutant that specifically affects the NLRP3 inflammasome activation without increasing other pro-inflammatory signals, will allow to more precisely ask the question if inflammasome activation by Mtb is a host protective response. A previous report that a *PknF* transposon mutant is attenuated for growth in mouse lungs seems to suggest that inflammasome inhibition might be important for virulence [52]. We would like to repeat and reproduce these results with our deletion mutant and the complemented strain. Also, we want to confirm that any *in vivo* attenuation is really due to the difference in inflammasome activation by comparing the wild-type and mutant Mtb strain virulence in *Nlrp3*^*-*/-^ mice.

How does Mtb PknF inhibit the NLRP3-inflammasome activation? PknF belongs to the 11-member family of serine/threonine protein kinases in Mtb [41]. It has a predicted transmembrane domain and no known secretion signal and thus remains associated with the Mtb cell membrane [41,53]. This fact makes a direct interaction of PknF with the host cell inflammasome activation pathway unlikely. PknF is capable of phosphorylating many Mtb proteins (at least in *in vitro* assays) [54] but the best described target is the ABC-like transporter protein Rv1747 [55,56]. Rv1747 is phosphorylated by PknF at two specific threonine residues which leads to its activation [56]. Rv1747 is important for virulence of Mtb since a deletion mutant is attenuated in mice [55]. Phosphatidyl-myo-innositol mannosides (PIMs) are a class of cell wall associated lipids that are transported via Rv1747 [57]. PIMs of Mtb have the capacity to affect host cell functions; for example, they have a role in inhibiting phagosome-lysosome fusion by stimulating fusing of the phagosome with early endosomes [58]. Mtb lipids in general are broadly distributed throughout the host cell organelles [51,59,60]. Our working model is thus that the lack of PknF leads to a decrease in Rv1747 activity which results in less secreted PIMs which are then unable to limit NLRP3-infammasome activation. The potential role of a lipid in the process of NLRP3-inflammasome inhibition is further supported by the fact that this inhibition is independent of the ESX-1 secretion system (Fig 1C and 1D) which is essential for any Mtb protein to reach the host cell cytosol but should not affect the distribution of Mtb lipids. The positive impact of IL-1β on host resistance to Mtb infections in the mouse model is well supported by several studies using *Il1r1*^-/-^ and *Il1b*^-/-^ knock-out mouse strains [3,4,6,26]. The protection of IL-1β is not dependent on cell-intrinsic defense mechanisms since wild-type cells of both hematopoietic and non-hematopoietic origin 1 restored host resistance in *Il1r1*^-/-^ deficiency *in vivo* [11]. An independent line of investigation supports a detrimental role for excessive IL-1β during Mtb infection. IFN-γ induced production of nitric oxide (NO) is able to suppress Il-1β production by inhibiting activation of the NLRP3-inflammasome, most likely via S-nitrosylation of NLRP3 which inhibits the assembly of the inflammasome complex [61]. The NO-mediated suppression of inflammasome activation is important to inhibit IL-1-dependent neutrophil recruitment during later stages of Mtb infections which is important for host resistance [62]. It thus is possible that the role of IL-1β is dependent on the spatial and temporal context during the infection with Mtb. Consequently, the capacity to modulate the extend of the host cell IL-1β-response could be of benefit to Mtb. The NLRP3-inflammasome activating *PknF* Mtb mutant will be an interesting tool to further investigate the complex role of IL-1β in the host response to Mtb.

## Materials and methods

### Ethics statement

The University of Maryland is fully accredited by the Association for Assessment and Accreditation of Laboratory Animal Care International (AAALAC). Work involving mice was approved by the University of Maryland IACUC (Protocol#R-MAR-19-12). The peripheral blood mononuclear cells were provided by anonymous donors in a protocol approved by the UMD Institutional Review Board (1316476–1).

### Bacterial strains and growth conditions

*M*. *tuberculosis* H37Rv and CDC1551 strains were grown in liquid Middlebrook 7H9 medium supplemented with 10% oleic acid-albumin-dextrose- catalase (OADC) growth supplement, 0.2% glycerol, and 0.05% tween 80 or on solid MB 7H11 agar containing 0.2% glycerol and supplemented with 10% OADC enrichment. For selection of *M*. *tuberculosis* deletion and complemented strains media was supplemented with following antibiotics: hygromycin 50 μg/ml and kanamycin 50 μg/ml respectively. For cloning and plasmid propagation, *Escherichia coli* DH5α, grown in Luria-Bertani (LB) medium or on LB agar at 37°C was used as a host strain. When required, LB media was supplemented with 50 μg/ml kanamycin. For preparing the heat-killed *M*. *tuberculosis* strains, bacteria were heat inactivated at 65°C or 80°C for 40 min.

### Generation of *M*. *tuberculosis pknF* (*MT1788*) deletion mutant and complemented strains

The *pknF* deletion mutant (Δ*pknF*) was generated in *M*. *tuberculosis* CDC1551 strain by using specialized phage transduction strategy as described previously [42]. Recombinants were selected on 7H11 agar plates containing the appropriate antibiotics. Gene deletion was confirmed by Southern blotting. Complementation of Δ*pknF* was achieved by amplifying the full length *pknF* (1431bp) gene sequence and cloning it into the integrative plasmid pMV361. This recombinant plasmid was electroporated into the Δ*pknF* deletion strain and recombinants were selected on 7H11 plates with 50μg/ml kanamycin and hence generated a complemented strain (Δ*pknF*::*pknF*) expressing *pknF* under the control of a constitutive promoter (Hsp60). The sequences of all primers used in this study are listed in S1 Table. The Mtb *ΔesxA* mutant and Δ*esxA*::*esxA* complemented *strains* were kindly provided by Dr. L. Gao and were created on the Mtb H37Rv background. The reporter expression construct pMV261-*rfp* was electroporated into Δ*pknF* mutant strain and pMV261-*gfp* was electroporated into both Mtb and Δ*pknF* mutant strain. After electroporation the recombinants were selected on 7H11 agar plates containing the appropriate antibiotics and thus generated the following reporter strains Mtb-*gfp*, Δ*pknF-gfp* and Δ*pknF-rfp* that were used for co-infection and co-localization experiments.

### *In vitro* growth kinetics of recombinant *M*. *tuberculosis* strains

Wild-type *M*. *tuberculosis (Mtb)*, Δ*pknF* deletion and Δ*pknF*::*pknF* complement CDC1551 strains were grown for 24 days at 37°C in Middlebrook 7H9 medium supplemented with 10% OADC, 0.2% glycerol, and 0.05% tween 80 and for selection of recombinant strains the following antibiotics were added at indicated concentrations: hygromycin 50 μg/ml and kanamycin 50 μg/ml. The cultures were diluted to an OD600 of 0.04 using Middlebrook 7H9 medium and then aliquots were removed from each culture at different time points and the OD600 was determined at 4, 8, 12, 16, 20 and 24 days of incubation at 37°C.

### Cell culture and infections

Bone marrow cells were aseptically flushed from the femurs and tibias of following mice: Wild-type (WT) C57BL/6J, NO synthase 2 (*Nos2*)^*−/−*^, *Nox2*^*-/-*^, *mCAT*, *Casp3*^*-/-*^, *Casp1*^*-/-*^ and *GsdmD*^*-/-*^ were obtained from Jackson Laboratories. *Nlrp3*^*-/-*^ and *Aim2*^*-/-*^ mice were provided by Dr. Vijay Rathinam. *Asc*^*-/-*^ mice were provided by Dr. Mahtab Moayeri. *Ripk3*^*-/-*^, *Ripk3*^*-/-*^/*Casp8*^*-/-*^ and *Trif*^*-/-*^*/MyD88*^*-/-*^ mice were provided by Dr. Igor Brodsky. *Casp11*^*-/-*^ and *Casp1/11*^*-/-*^ mice were provided by Dr. Denise Monack. Bone marrows cells were cultured for 7 days in Dulbecco’s modified eagle medium (DMEM) containing 10% heat-inactivated FBS, 20% L929 conditioned medium, 1% penicillin/streptomycin at 37°C and 5% CO_2_ to allow differentiation into macrophages. After differentiation bone marrow-derived macrophages (BMDMs) were harvested, plated into 24 or 96, well plates (5 × 10^5^, 4 × 10^4^ cells per well respectively) in antibiotic-free BMDM media and were allowed to adhere and rest for 24 h just before the day of infection. On the day of infection media was removed, cells were washed twice with pre-warmed 1x PBS. BMDM were then infected with *Mtb*, and recombinant *Mtb* strains (Δ*pknF*, Δ*pknF*::*pknF*, Δ*esxA* and Δ*esxA*::*esxA*) in DMEM supplemented with 10% FBS and 20% L929 conditioned media at a multiplicity of infection (MOI) of 10:1. Infected macrophages were maintained at 37°C in a humidified atmosphere with 5% CO_2._ After a 4-h phagocytosis period, infected BMDM were gently washed four times with pre-warmed 1x PBS before replacing with BMDM chase media containing DMEM supplemented with 10% FBS, 20% L929 conditioned media and 10μg/ml Gentamicin.

For co-infection experiments, BMDMs (5 × 10^5^), were seeded on glass coverslips in a 24-well plates in DMEM supplemented with 10% FBS, 20% L929 conditioned media and were allowed to adhere overnight. The next day cells were mono-infected at two different MOI (10:1 and 20:1) and co-infected at a MOI of 10:1 for each strain for 4 h with GFP labelled *Mtb* and RFP labelled Δ*pknF* strains and incubated at 37°C with 5% CO2. After 4 h co-infection, coverslips were washed with 1x PBS and fixed with 4% paraformaldehyde. After fixation, cells were washed with 1x PBS before mounting in ProLong antifade mounting medium with DAPI (Invitrogen). Co-infected cells were then imaged on Zeiss LSM 980 confocal Laser scanning microscope equipped with a 63X oil-immersion objective. Cell culture supernatants were harvested at 20 hpi and analyzed for cell death and IL-1β secretion.

Peripheral blood mononuclear cells (PBMCs) from healthy blood donors were isolated from leukopaks by using Ficoll density gradient centrifugation. Then, monocytes were isolated from PBMCs by plastic adherence method. Cells were allowed to adhere in T25 cell culture flasks for 2 h at 37°C with 5% CO_2._ Non-adherent cells were washed away and the adherent monocytes were differentiated into macrophages using 10 ng/ml hM-CSF (R&D Systems) for 7 days in RPMI1640 containing 5% off-clot human serum (Gemini), 1% penicillin/streptomycin at 37°C and 5% CO_2._ Human monocyte derived macrophages (hMDMs) were then infected at day 7 at a MOI of 10:1 with *Mtb*, and recombinant *Mtb* strains (Δ*pknF and* Δ*pknF*::*pknF*) in RPMI1640 supplemented with 10% human AB serum.

For all experiments, the time point designated as “0 hpi” refers to immediately after 4 h of infection period, while “20 hpi” refers to the 20-h chase period after 4 h of infection. Cell culture supernatants and lysates were collected at desired post-infection time points for further analysis.

### Cell culture treatments

BMDMs (5 × 10^5^), were seeded in 24-well plates in DMEM supplemented with 10% FBS, 20% L929 conditioned media. When required, cells were treated with the following reagents: increasing concentrations of extracellular KCl (0mM, 10mM, 20mM, 40mM), chloride channel blockers DIDS (100μM, Tocris Bioscience) and NPPB (100μM, Tocris Bioscience), ROS inhibitors NAC (10mM, Sigma-Aldrich) and Tempol (100μM, R&D Systems), Caspase-3 inhibitor Z-DEVD-FMK (20 μM, R&D Systems), Xanthine oxidase inhibitor allopurinol (250μg/ml, MP Biomedicals), and calcium chelator BAPTA-AM (30μM, Millipore Sigma) either before infection with *Mtb*, Δ*pknF* mutant, and Δ*pknF*::*pknF* strains or before stimulation with NLRP3 inflammasome activators (Nigericin, 20μM, InvivoGen and ATP, 5mM, Thermo Scientific) for 30 min. Uninfected samples without any treatment were used as a control in the experiment. Cell culture supernatants were harvested at indicated time points and analyzed for IL-1β secretion by ELISA.

### Evaluation of NLRP3 inflammasome inhibition by *M*. *tuberculosis* in BMDMs

BMDMs (5 × 10^5^), were seeded in 24-well plates in DMEM supplemented with 10% FBS, 20% L929 conditioned media and incubated overnight at 37°C with 5% CO2. BMDMs were primed for 4 h with 100 ng/ml LPS and simultaneously infected with *Mtb* and recombinant *Mtb* strains (Δ*pknF*, Δ*pknF*::*pknF*, Δ*esxA* and Δ*esxA*::*esxA*) before stimulation with NLRP3 inflammasome activators (Nigericin, 1μM and ATP, 0.1mM) for 30 min. Uninfected samples treated with LPS and NLRP3 stimulators were used as a control in the experiment. Cell free supernatants were harvested immediately after 30 min stimulation and analyzed for IL-1β secretion and cell death.

### Detection of ASC speck formation by fluorescence microscopy

BMDMs were seeded on glass coverslips in a 24 well plate overnight. The next day cells were primed with 100 ng/ml LPS for 4 h. Concurrently, BMDM were infected for 4 h with *Mtb* and recombinant *Mtb* strains (Δ*pknF*, Δ*pknF*::*pknF*, Δ*esxA* and Δ*esxA*::*esxA*) and incubated at 37°C with 5% CO2. Four hours post-infection, cells were stimulated with NLRP3 agonists: 0.1 mM ATP for 30 min. Following stimulation, media from each well was aspirated, fixed with 4% paraformaldehyde. Before staining, cells were washed with 1x PBS and blocked with 0.2% BSA-0.05% saponin in 1x PBS for 30 min at room temperature. Cells were incubated overnight with primary antibody (ASC/TMS1 [D2W8U] Rabbit mAb Mouse Specific Cat# 67824S, Cell Signaling Technology) in blocking buffer at 4°C, followed by incubation with secondary antibody (Alexa Fluor 594 AffiniPure Donkey Anti-Rabbit IgG Cat# 711-585-152, Jackson Immunoresearch) for 1 h at room temperature. Bacteria were imaged by taking advantage of their autofluorescence by using excitation wavelength of 436/20nm and emission filter 480/40nm. Cells were washed three times with 1x PBS before mounting in ProLong antifade mounting medium with DAPI and imaged on Keyence fluorescence microscope. Images were analyzed using the Image J software.

### Immunofluorescence staining and Colocalization studies

BMDMs were seeded onto glass coverslips at a density of 2 × 10^5^ cells per well in 24 well plates just before the day of infection. The next day cells were infected for 4 h with GFP-expressing Mtb and Δ*pknF* strains at a MOI of 10:1. At 6 hpi media was aspirated from each well and cells were fixed with 4% paraformaldehyde. Fixed cells were washed with PBS and blocked with 5% non-fat milk and 0.1% saponin in 1x PBS for 30 min at room temperature. Coverslips were then incubated overnight with primary antibody (α-TAX1BP1 A303-791A; 1:300; Bethyl Laboratories) diluted in blocking buffer at 4°C. Coverslips were washed with PBS and incubated with secondary antibody (Alexa Fluor 647 AffiniPure Goat Anti-Rabbit IgG Cat# 111-605-045, Jackson Immunoresearch) for 1 h at room temperature. Cells were washed with PBS before mounting in ProLong antifade mounting medium with DAPI. Cells were then imaged on Zeiss LSM 980 confocal Laser scanning microscope equipped with a 63X oil-immersion objective. Confocal images were analyzed using ImageJ software. Colocalization between TAX1BP1 and Mtb strains was quantified in 36 randomly selected fields of view from each strain that included 4 to 5 cells per field in three independent experiments by determining the Pearson’s correlation coefficient (r) with ImageJ JaCoP plug-in (A value of -1 indicates perfect exclusion, zero represents random localization, while +1 indicates perfect correlation).

### Cell death assay and Caspase-1 activity assay

Cell death was monitored with the ToxiLight BioAssay Kit (Cat# LT17-217, Lonza) as per the manufacturer’s instructions. This assay measures the release of adenylate kinase (AK) enzyme in cell supernatant upon damage to the plasma membrane.

Caspase-Glo 1 Inflammasome Assay, a bioluminescent method was used for measuring the Caspase-1 activity in cell culture supernatant (Cat# G9951, Promega). Briefly, BMDMs (4 × 10^4^) were seeded per well into 96-well plate and infected with *Mtb*, Δ*pknF* and Δ*pknF*::*pknF* strains and cell supernatant was collected at 20 hpi. The Caspase-Glo 1 Inflammasome assay was performed according to the manufacturer’s instructions. Caspase-Glo 1 reagent was added to 96-well plate containing the cell free supernatants from infected and uninfected cells. Cells were incubated for 90 min and the luminescence was measured by Synergy HTX multimode plate reader (BioTek).

### Cytokine detection by enzyme-linked immunosorbent assay (ELISA)

ELISA was used to measure secreted IL-1β, IL-18, IL-6 and IFNβ in cell-free supernatants harvested at indicated time points using R & D Systems Mouse/Human IL-1 beta/IL-1F2 DuoSet ELISA kit, Mouse IL-18 DuoSet ELISA kit, Mouse IL-6 DuoSet ELISA kit and Mouse IFNβ DuoSet ELISA kit respectively. The assay was performed according to manufacturer’s instruction. For determining the cytokine concentration of the samples in 96-well plate absorbance was measured at 450 nm and calculated by GraphPad Prism linear regression analysis.

### Immunoblotting

Whole-cell lysates were obtained by lysing BMDMs with NP-40 lysis buffer supplemented with protease inhibitor and a phosphatase inhibitor cocktail. Protein concentration was measured by a BCA Protein Assay Kit (#23225, Thermo Scientific). Cell lysates were subjected to SDS-PAGE and proteins were transferred onto nitrocellulose membrane. The membrane was blocked at room temperature for 2 h in blocking buffer containing 5% non-fat milk/TBS-Tween. After blocking, membrane was washed three times with TBS-Tween and incubated with primary antibodies overnight at 4°C. For this study, the following primary antibodies were used: Goat Anti-Mouse IL-1β/IL-1F2 Antigen Affinity-purified Polyclonal Antibody (# AF-401-NA, R&D Systems) and Beta Actin Monoclonal antibody (# 66009-1-Ig, Proteintech). The membrane was washed, followed by incubation at room temperature for 1 h with HRP-conjugated Anti-Goat and Anti-mouse secondary antibodies. After washing, the protein bands were visualized with SuperSignal West Femto chemiluminescent substrate (#34095, Thermo Scientific) following the manufacturer’s instructions. Protein bands were quantified with Image J software.

### Calcium mobilization assay

Mobilization of intracellular calcium was detected by Fluo-Forte Calcium Assay kit (#ENZ-51017, Enzo Life Sciences) that utilizes a fluorogenic calcium-binding dye that binds to intracellular calcium and thereby producing a fluorescence signal. The assay was performed as per the manufacturer instructions. Briefly, BMDMs (4 × 10^4^ cells per well) were seeded in BMDM growth medium in 96- well plates and infected with *Mtb*, Δ*pknF* and Δ*pknF*::*pknF* strains. Fluo-Forte Dye-Loading Solution was added to each well followed by incubation for 1 hour at RT and calcium flux was monitored at 4 h and 6 h post infection time points. For this study, uninfected cells treated with LPS (1μg/ml) for 4 h and stimulated with NLRP3 agonist (ATP, 5mM) for 30 min were used as a positive control. Fluorescence was monitored at Ex = 490 nm/Em = 525 nm with Synergy HTX multimode plate reader (BioTek).

### Measurement of intracellular levels of potassium

The intracellular levels of K^+^ was measured by the FluxOR II green potassium ion channel assay (#F20016, Invitrogen) as previously described [63]. The assay was performed following the manufacturer’s protocol. For experiment control, BMDMs were primed with LPS (1μg/ml) for 4 h, and stimulated with ATP (0.1mM) for 30 min. Briefly, BMDMs (2 × 10^5^ cells/ml) were seeded in 96-well plates and infected with *Mtb*, Δ*pknF* and Δ*pknF*:*pknF* strains for 4 h. A potassium channel stimulus buffer was added to cells at 2 h and 6 h post infection time points and the intracellular fluorescence was measured at Ex = 490 nm/Em = 525 nm with Synergy HTX multimode plate reader (BioTek).

### ROS detection and Xanthine oxidase activity assay

Intracellular levels of ROS was measured at 6 hpi in both uninfected and infected BMDM in the presence or absence of ROS scavenger NAC using CellROX Green Reagent (#C10444, Thermo Scientific) according to the manufacturer’s instructions. A Biotek Synergy HTX multimode plate reader was used for fluorescence measurements of CellROX Green with excitation/emission maxima of ~485/520 nm. Xanthine oxidase enzyme activity was measured by Xanthine Oxidase Activity Assay Kit (#MAK078, Sigma-Aldrich). The assay was performed as described in the manufacturer’s protocols.

### Cathepsin B activity assay

Intracellular Cathepsin B activity was measured at specified time points in infected BMDM using a Magic Red Cathepsin B assay Kit according to the manufacturer’s instructions (#937, Immunochemistry).

### Mycobacterial total lipid extraction and TLC analysis

Mycobacterial total lipids were extracted and analyzed by one-dimensional TLC as described previously [64,65]. Briefly, *Mtb*, Δ*pknF* mutant and Δ*pknF*::*pknF* strains were grown in Middlebrook 7H9 medium to an OD_600_ of 0.8. Bacterial cultures were harvested and pellet was resuspended in methanol:chloroform (2:1). After overnight incubation samples were centrifuged and supernatant was collected. Pellet was suspended in methanol:chloroform (1:1). After an hour, samples were centrifuged and supernatant was collected and pooled with the first fraction. The extraction was repeated again with methanol:chloroform (1:2) and supernatant collected at this step is pooled with the other two fractions. Lipids were dried and 100μg of the total lipid extract from each sample was spotted onto TLC plates using glass micro-capillary pipettes and subjected to one-dimensional TLC analysis. PDIMs were developed using petroleum ether:diethyl ether (9:1) and lipid spots were visualized by staining with 5% solution of molybdophosphoric acid in 95% ethanol followed by charring at 140°C.

### Statistical analysis

Statistical comparisons were made using ordinary one-way ANOVA multiple comparisons with Prism 8.4.2 (GraphPad). All experiments were performed at least three times and experimental values were reported as the means ± SEM/SD. Data are plotted as “SuperPlots” that display both experimental robustness and cell to cell variability. Each colored data point in graph represents average of technical replicates for each of the biological replicate and therefore address the reproducibility of the findings.

## Supporting information

S1 Fig Nigericin and ATP treatment induces the secretion of IL-1β in a dose-dependent manner in BMDMs. BMDMs were either left untreated (UT) or treated with 100ng/ml LPS for 4 h and then stimulated for 30 min with two different NLRP3 inflammasome activators, Nigericin and ATP at indicated doses. Cell supernatants were harvested after 30 min of stimulation and analyzed for (A, B) IL-1β release by ELISA. Data are representative of three independent experiments.
(TIF)

S2 Fig Mtb infection leads to significant decrease in the number of ASC specks-positive cells which is independent of the ESX-1 secretion system. BMDMs were primed with LPS (100ng/ml) for 4 h and simultaneously infected with different H37Rv Mtb strains (Mtb, Δ*esxA*, and Δ*esxA*::*esxA***)** or left uninfected (UI) followed by treatment with the NLRP3 activator ATP (0.1mM) for 30 min. Cells were fixed, permeabilized and immunostained for ASC (Alexa Fluor 594, red). ASC specks were detected by fluorescence microscopy. Scale bar 10μm. Bacteria were detected using their autofluorescence (Excitation 436/20 nm; Emission 480/40 nm) Data are representative of three independent experiments.
(TIF)

S3 Fig Generation and characterization of Mtb *pknF* deletion mutant strain. (A) Confirmation of *pknF* deletion mutant strain constructed in Mtb CDC1551 by Southern blot analysis: Lane 1, WT Mtb; Lane 2, *ΔpknF* mutant. (B) In vitro growth analysis of Mtb, *ΔpknF* mutant and complement Δ*pknF*::*pknF* in 7H9 medium. (C) Comparison of PDIM levels in Mtb, *ΔpknF* mutant and complement Δ*pknF*::*pknF* by one dimensional TLC analysis. Data are representative of three independent experiments.
(TIF)

S4 Fig *pknF* Mtb mutant infection induces ASC speck formation followed by increased production of inflammasome dependent cytokine IL-18. BMDMs were either left uninfected (UI) or infected with different CDC1551 Mtb strains (Mtb, *ΔpknF* mutant and complement Δ*pknF*::*pknF*) at an MOI of 10 for 4h. (A) The culture supernatants were harvested at 0 hpi, 6 hpi and 20 hpi and analyzed for secretion of IL-18 by ELISA. At 6 hpi (B) cells were fixed, permeabilized and immunostained for ASC (Alexa Fluor 594, red) and ASC specks were detected by fluorescence microscopy, scale bar 20μm, insets are enlargements of the boxed regions. Data are representative of three independent experiments. Error bars represent mean ± SEM; *, p<0.05, **, p<0.01.
(TIF)

S5 Fig Live *pknF* Mtb mutant is required to induce NLRP3 inflammasome activation in BMDMs. BMDMs were either left uninfected (UI) or infected with live or heat killed (65°C or 80°C for 30 min) or chloramphenicol (30μg/ml) treated CDC1551 Mtb wild-type and *ΔpknF* mutant at an MOI of 10 for 4h. The cell culture supernatants were harvested at 20 hpi and assessed for (A) IL-1β secretion by ELISA and (B) cell death by measuring the release of adenylate kinase (AK). Data are representative of three independent experiments. Error bars represent mean ± SEM; *, p<0.05, **, p<0.01, ***, p<0.001, ****, p<0.0001, ns (non-significant).
(TIF)

S6 Fig Increased production of IL-1β and cell death in BMDMs infected with *pknF* Mtb mutant is independent of AIM2 inflammasome and Ripk3/Caspase 8 complex. BMDMs derived from wild type (WT), *Aim2*^-/-^, *Ripk3*^-/-^, and *Ripk3*^-/-^*/Casp8*^*-*/-^ mice were either left uninfected (UI) or infected with CDC1551 Mtb and *ΔpknF* mutant at an MOI of 10 for 4h. The culture supernatants were harvested at 20 hpi and analyzed for (A, B) Secretion of IL-1β by ELISA and (C, D) cell death by quantification of the release of adenylate kinase (AK). Data are representative of three independent experiments. Error bars represent mean ± SEM; *, p<0.05, **, p<0.01, ns (non-significant).
(TIF)

S7 Fig *pknF* Mtb mutant infection compared to Mtb infection does not induce increase phagosomal membrane rupture and/or cytosolic access in BMDM. BMDMs were infected with CDC1551 Mtb and *ΔpknF* mutant at an MOI of 10 for 4h. At 6 hpi, cells were fixed, permeabilized and immunostained for TAX1BP1. Colocalization between TAX1BP1 and different Mtb strains (Mtb and *ΔpknF* mutant) was (A) imaged using LSM 980 Laser scanning confocal microscope, Scale bar 5μm and (B) quantified by determining the Pearson’s correlation coefficient (r) with ImageJ JaCoP plug-in in 36 randomly selected fields of view from each Mtb strain that included 4 to 5 cells per field in three independent experiments (A value of -1 indicates perfect exclusion, zero represents random localization, while +1 indicates perfect correlation). BMDMs derived from wild type (WT) and *Asc*^*-*/-^ mice were either left uninfected (UI) or infected with Mtb and *ΔpknF* mutant at an MOI of 10 for 4h. The culture supernatants were harvested at 20 hpi and analyzed for (C) IFN-β levels by ELISA. Data are representative of three independent experiments. Error bars represent mean ± SEM; **, p<0.01, ***, p<0.001, ns (non-significant).
(TIF)

S8 Fig Caspase 3 is partially responsible for the increased production of IL-1β and cell death in *pknF* Mtb mutant infected BMDMs. BMDMs derived from wild type (WT) and *Gsdmd*^*-*/-^ mice were either left uninfected (UI) or infected with CDC1551 Mtb and *ΔpknF* mutant at an MOI of 10 for 4h in the presence or absence of Caspase 3 inhibitor Z-DEVD-FMK. The culture supernatants were harvested at 20 hpi and analyzed for (A) cell death and (B) IL-1β levels by AK assay and ELISA respectively. BMDMs derived from WT and *Casp3*^*-*/-^ mice were either left uninfected (UI) or infected with Mtb and *ΔpknF* mutant at an MOI of 10 for 4h. The culture supernatants were harvested at 20 hpi and analyzed for (C) cell death and (D) IL-1β levels by AK assay and ELISA respectively. Data are representative of three independent experiments. Error bars represent mean ± SEM; *, p<0.05, **, p<0.01, ***, p<0.001, ns (non-significant).
(TIF)

S9 Fig NLRP3 inflammasome activation induced by the *pknF* Mtb mutant is dependent on chloride efflux but independent of calcium influx. BMDMs were either left untreated or treated with chloride channel blocker, DIDS (100μM) or with BAPTA-AM (30μM), Ca^2+^ chelator and then infected with CDC1551 Mtb wild-type and *ΔpknF* mutant. Culture supernatants were harvested at 20 hpi and analyzed for (A, B) IL-1β secretion by ELISA. BMDMs were either left uninfected (UI) or infected with different CDC1551 Mtb strains (Mtb, *ΔpknF* mutant and complement Δ*pknF*::*pknF*) at an MOI of 10 for 4h. BMDMs were primed with LPS (1μg/ml) for 4 h and stimulated with ATP (5mM, 30 min) and used as positive control for inducing NLRP3 inflammasome activation. Intracellular calcium mobilization was analyzed at (C) 4 hpi and (D) 6 hpi by Fluo-Forte Calcium Assay kit. Data are representative of three independent experiments. Error bars represent mean ± SEM; **, p<0.01, ****, p<0.0001, ns (non-significant).
(TIF)

S10 Fig *pknF* Mtb mutant induced NLRP3 inflammasome activation is dependent on ROS but not on the release of Cathepsin B. BMDMs were either left untreated (-Tempol) or treated with ROS scavenger, Tempol (100μM) and then infected with CDC1551 Mtb wild-type and *ΔpknF* mutant at an MOI of 10 for 4h. Culture supernatants were harvested at 20 hpi and analyzed for (A) IL-1β secretion by ELISA. BMDMs were either left untreated (UT) or treated with 1μg/ml LPS for 4 h and then stimulated with two different NLRP3 inflammasome activators, Nigericin (20μM) and ATP (5mM) for 30 min. When required, BMDMs were treated with allopurinol (250μg/ml), xanthine oxidase inhibitor before stimulation with Nigericin/ATP. Cell supernatants were harvested after 30 min of stimulation and analyzed for (B) IL-1β release by ELISA. BMDMs were either left uninfected (UI) or infected with different CDC1551 Mtb strains (Mtb, *ΔpknF* mutant and complement Δ*pknF*::*pknF*). (C) Intracellular cathepsin B activity was monitored at 0, 2, 4, 6, 20 and 24 hpi by Magic Red Cathepsin B assay Kit Data are representative of three independent experiments. Error bars represent mean ± SEM; *, p<0.05, ****, p<0.0001, ns (non-significant).
(TIF)

S1 Table Primers used in this study. (DOCX)
