# Supplementary figures and images for: Mycobacterium tuberculosis inhibits the NLRP3 inflammasome activation via its phosphokinase PknF
Source: PLoS Pathog. 2021 Jul 29;17(7):e1009712. doi: 10.1371/journal.ppat.1009712 (PMC8321130; doi:10.1371/journal.ppat.1009712)

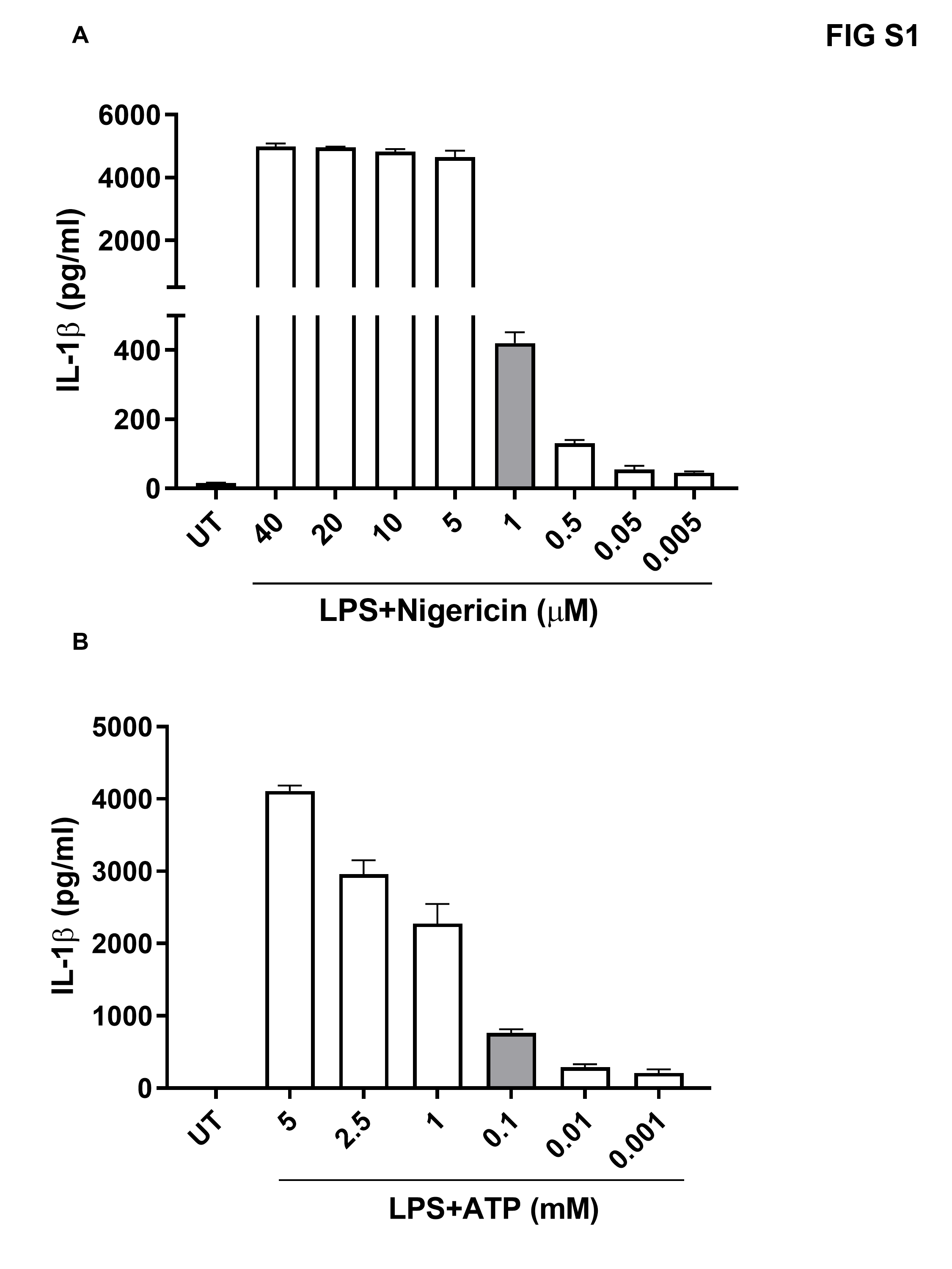

Supplement: S1 Fig — BMDMs were either left untreated (UT) or treated with 100ng/ml LPS for 4 h and then stimulated for 30 min with two different NLRP3 inflammasome activators, Nigericin and ATP at indicated doses. Cell supernatants were harvested after 30 min of stimulation and analyzed for (A, B) IL-1β release by ELISA. Data are representative of three independent experiments. (TIF) [file ppat.1009712.s001.tif]

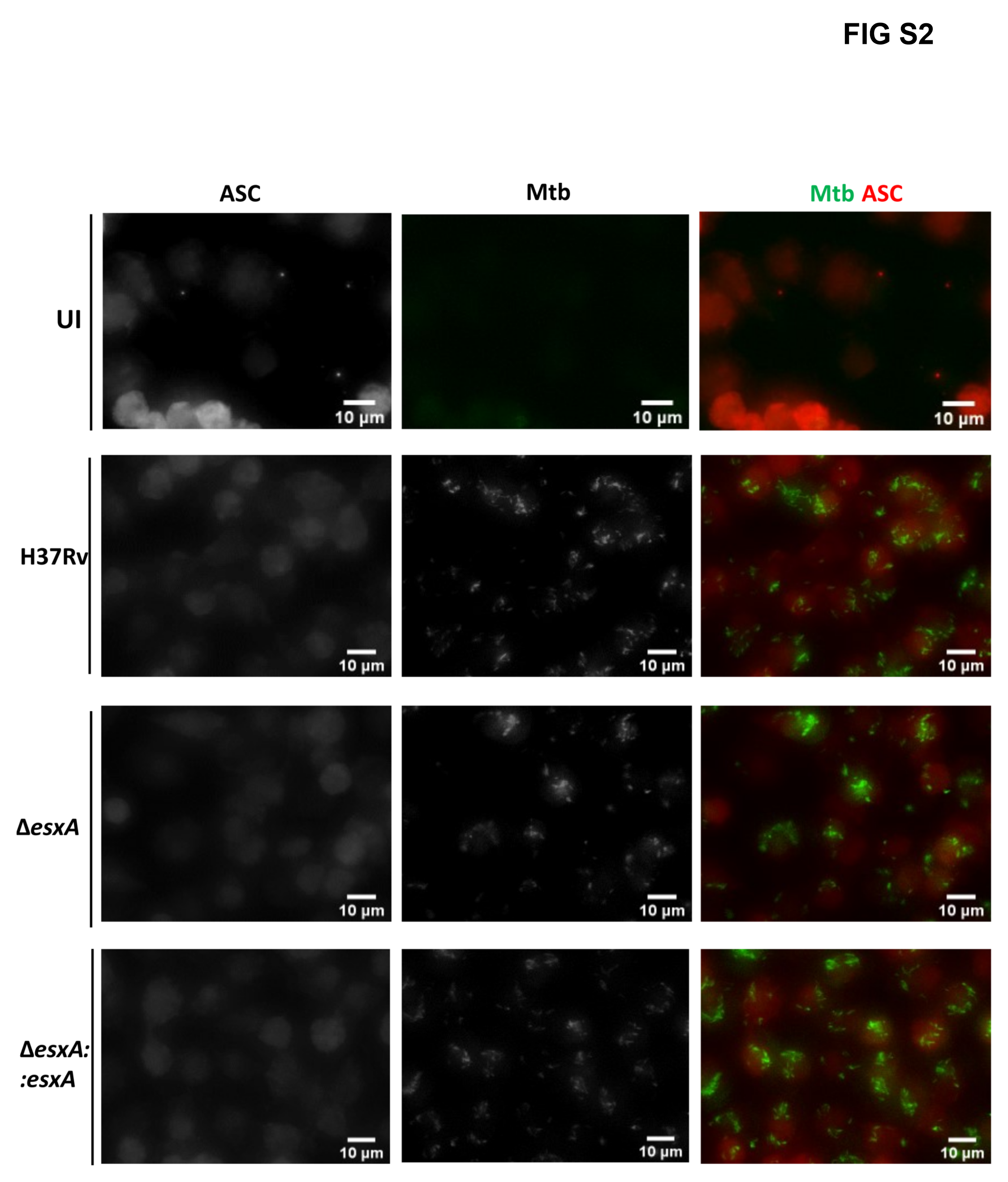

Supplement: S2 Fig — BMDMs were primed with LPS (100ng/ml) for 4 h and simultaneously infected with different H37Rv Mtb strains (Mtb, ΔesxA, and ΔesxA::esxA) or left uninfected (UI) followed by treatment with the NLRP3 activator ATP (0.1mM) for 30 min. Cells were fixed, permeabilized and immunostained for ASC (Alexa Fluor 594, red). ASC specks were detected by fluorescence microscopy. Scale bar 10μm. Bacteria were detected using their autofluorescence (Excitation 436/20 nm; Emission 480/40 nm) Data are representative of three independent experiments. (TIF) [file ppat.1009712.s002.tif]

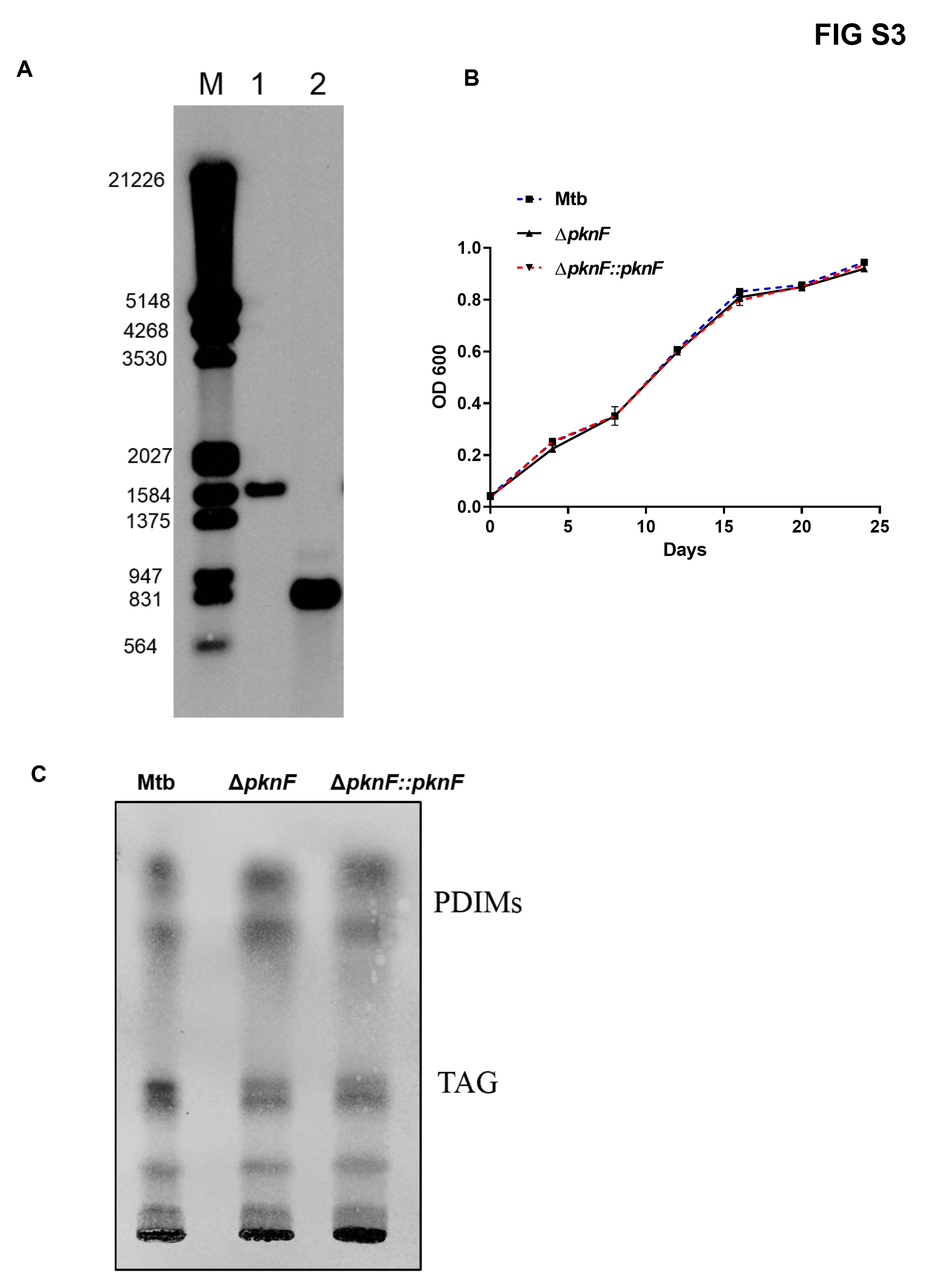

Supplement: S3 Fig — (A) Confirmation of pknF deletion mutant strain constructed in Mtb CDC1551 by Southern blot analysis: Lane 1, WT Mtb; Lane 2, ΔpknF mutant. (B) In vitro growth analysis of Mtb, ΔpknF mutant and complement ΔpknF::pknF in 7H9 medium. (C) Comparison of PDIM levels in Mtb, ΔpknF mutant and complement ΔpknF::pknF by one dimensional TLC analysis. Data are representative of three independent experiments. (TIF) [file ppat.1009712.s003.tif]

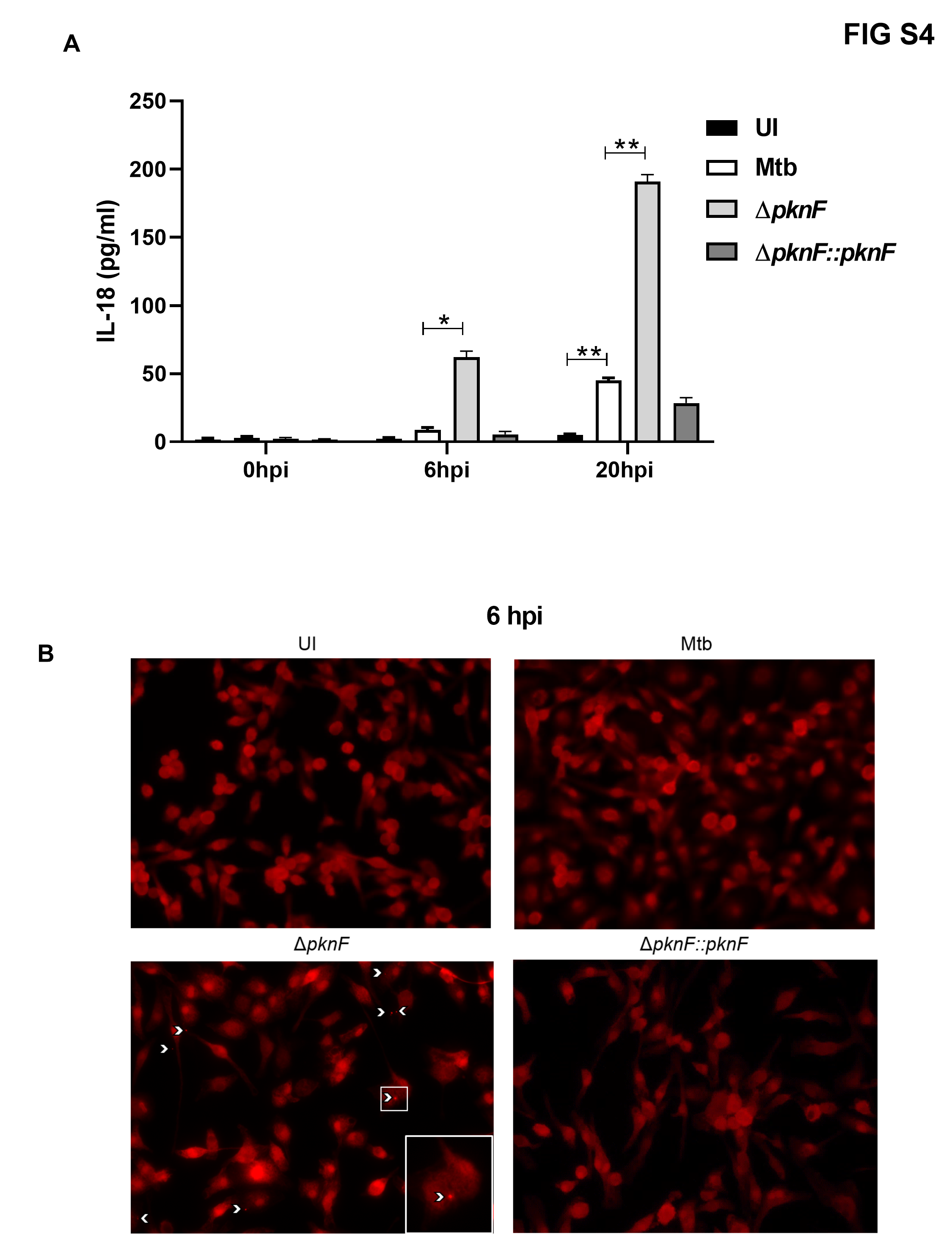

Supplement: S4 Fig — BMDMs were either left uninfected (UI) or infected with different CDC1551 Mtb strains (Mtb, ΔpknF mutant and complement ΔpknF::pknF) at an MOI of 10 for 4h. (A) The culture supernatants were harvested at 0 hpi, 6 hpi and 20 hpi and analyzed for secretion of IL-18 by ELISA. At 6 hpi (B) cells were fixed, permeabilized and immunostained for ASC (Alexa Fluor 594, red) and ASC specks were detected by fluorescence microscopy, scale bar 20μm, insets are enlargements of the boxed regions. Data are representative of three independent experiments. Error bars represent mean ± SEM; *, p<0.05, **, p<0.01. (TIF) [file ppat.1009712.s004.tif]

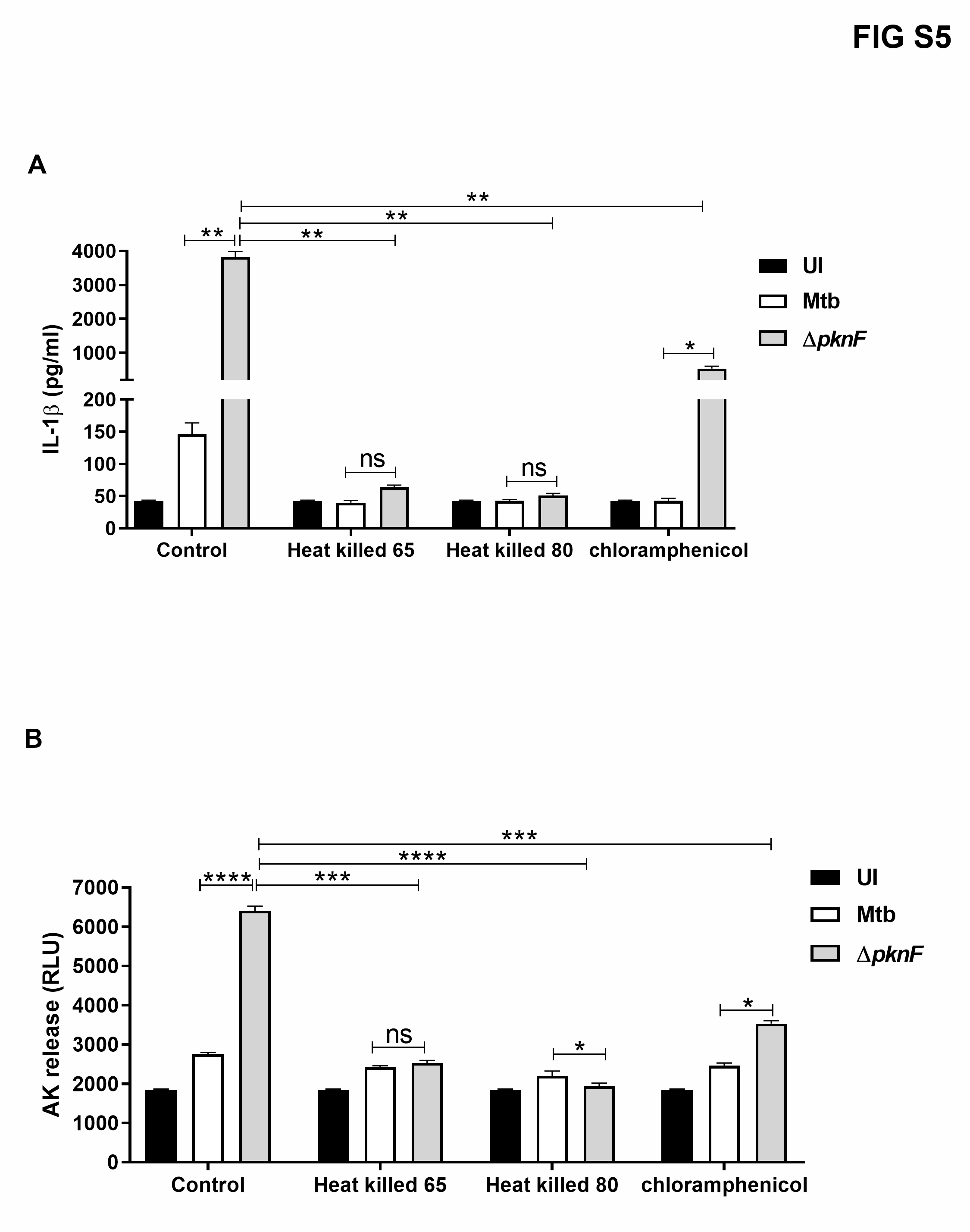

Supplement: S5 Fig — BMDMs were either left uninfected (UI) or infected with live or heat killed (65°C or 80°C for 30 min) or chloramphenicol (30μg/ml) treated CDC1551 Mtb wild-type and ΔpknF mutant at an MOI of 10 for 4h. The cell culture supernatants were harvested at 20 hpi and assessed for (A) IL-1β secretion by ELISA and (B) cell death by measuring the release of adenylate kinase (AK). Data are representative of three independent experiments. Error bars represent mean ± SEM; *, p<0.05, **, p<0.01, ***, p<0.001, ****, p<0.0001, ns (non-significant). (TIF) [file ppat.1009712.s005.tif]

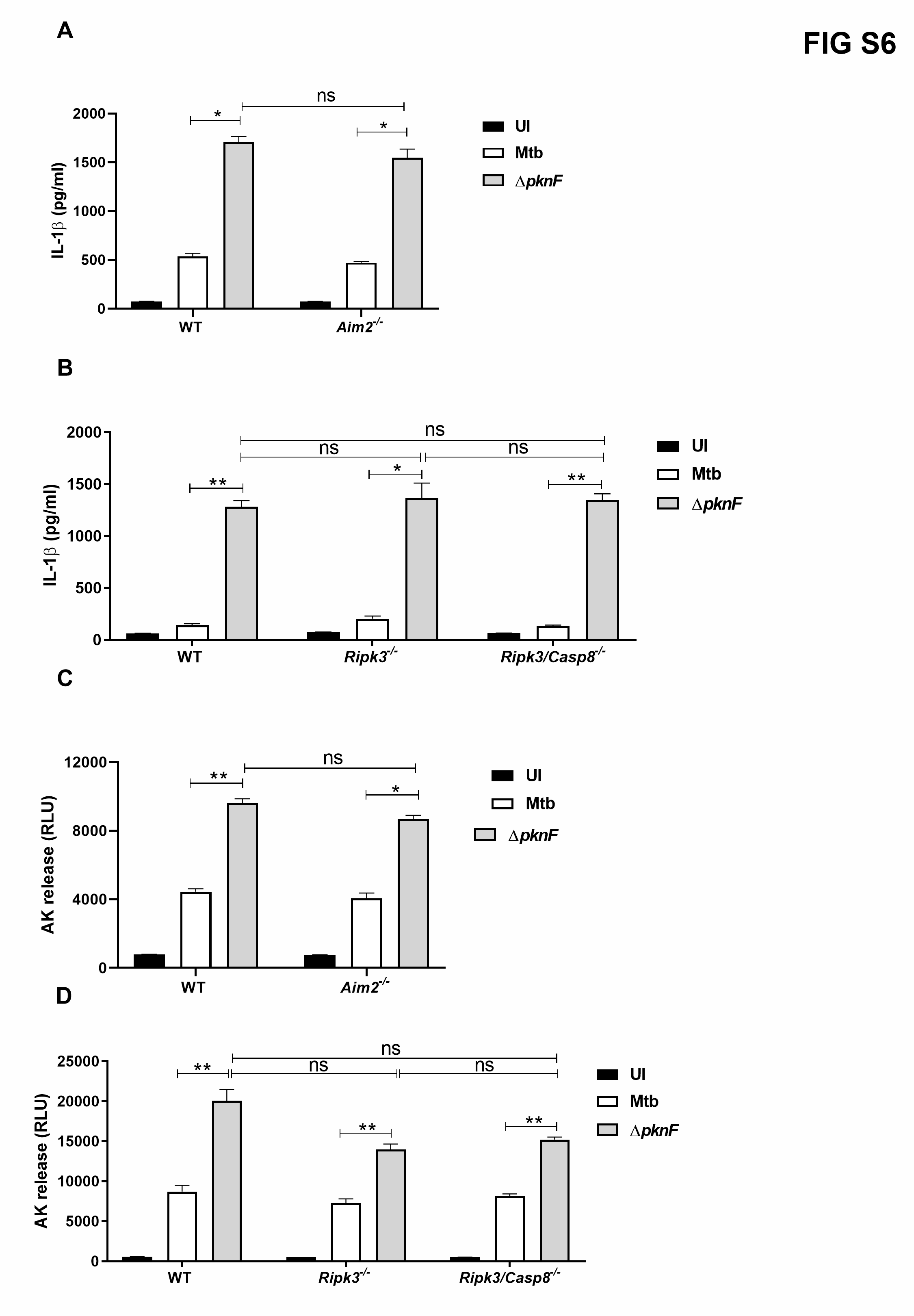

Supplement: S6 Fig — BMDMs derived from wild type (WT), Aim2-/-, Ripk3-/-, and Ripk3-/-/Casp8-/- mice were either left uninfected (UI) or infected with CDC1551 Mtb and ΔpknF mutant at an MOI of 10 for 4h. The culture supernatants were harvested at 20 hpi and analyzed for (A, B) Secretion of IL-1β by ELISA and (C, D) cell death by quantification of the release of adenylate kinase (AK). Data are representative of three independent experiments. Error bars represent mean ± SEM; *, p<0.05, **, p<0.01, ns (non-significant). (TIF) [file ppat.1009712.s006.tif]

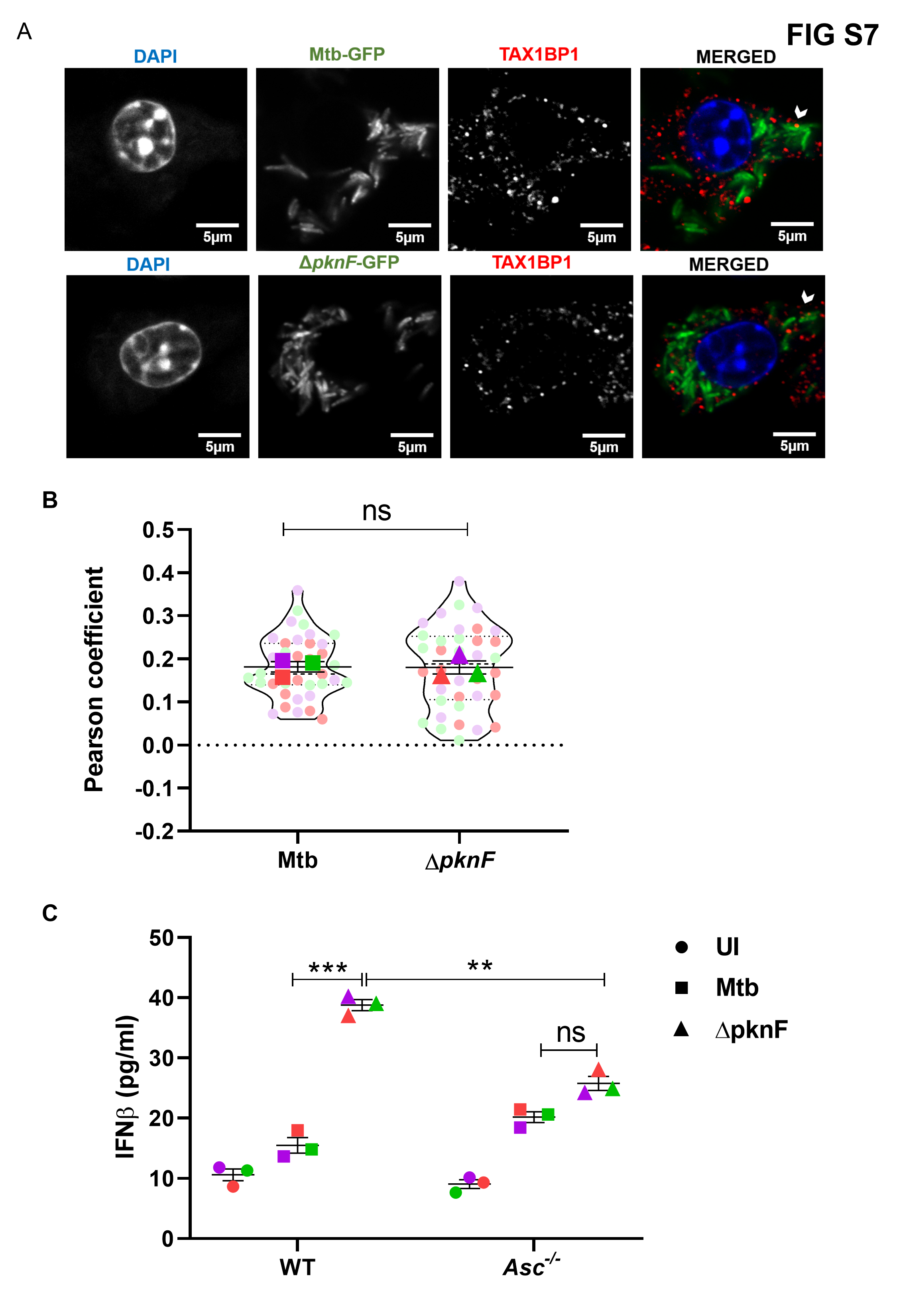

Supplement: S7 Fig — BMDMs were infected with CDC1551 Mtb and ΔpknF mutant at an MOI of 10 for 4h. At 6 hpi, cells were fixed, permeabilized and immunostained for TAX1BP1. Colocalization between TAX1BP1 and different Mtb strains (Mtb and ΔpknF mutant) was (A) imaged using LSM 980 Laser scanning confocal microscope, Scale bar 5μm and (B) quantified by determining the Pearson’s correlation coefficient (r) with ImageJ JaCoP plug-in in 36 randomly selected fields of view from each Mtb strain that included 4 to 5 cells per field in three independent experiments (A value of -1 indicates perfect exclusion, zero represents random localization, while +1 indicates perfect correlation). BMDMs derived from wild type (WT) and Asc-/- mice were either left uninfected (UI) or infected with Mtb and ΔpknF mutant at an MOI of 10 for 4h. The culture supernatants were harvested at 20 hpi and analyzed for (C) IFN-β levels by ELISA. Data are representative of three independent experiments. Error bars represent mean ± SEM; **, p<0.01, ***, p<0.001, ns (non-significant). (TIF) [file ppat.1009712.s007.tif]

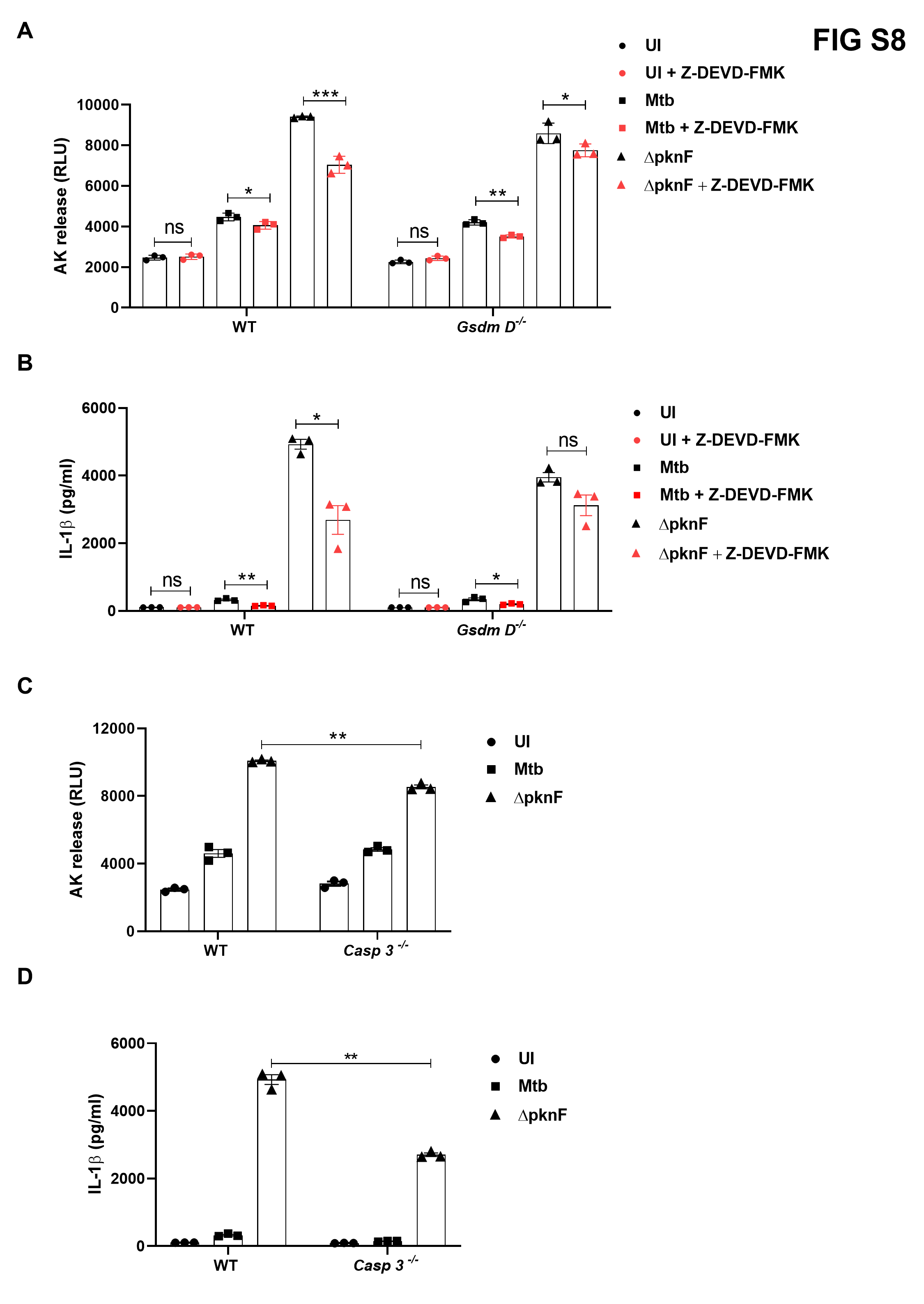

Supplement: S8 Fig — BMDMs derived from wild type (WT) and Gsdmd-/- mice were either left uninfected (UI) or infected with CDC1551 Mtb and ΔpknF mutant at an MOI of 10 for 4h in the presence or absence of Caspase 3 inhibitor Z-DEVD-FMK. The culture supernatants were harvested at 20 hpi and analyzed for (A) cell death and (B) IL-1β levels by AK assay and ELISA respectively. BMDMs derived from WT and Casp3-/- mice were either left uninfected (UI) or infected with Mtb and ΔpknF mutant at an MOI of 10 for 4h. The culture supernatants were harvested at 20 hpi and analyzed for (C) cell death and (D) IL-1β levels by AK assay and ELISA respectively. Data are representative of three independent experiments. Error bars represent mean ± SEM; *, p<0.05, **, p<0.01, ***, p<0.001, ns (non-significant). (TIF) [file ppat.1009712.s008.tif]

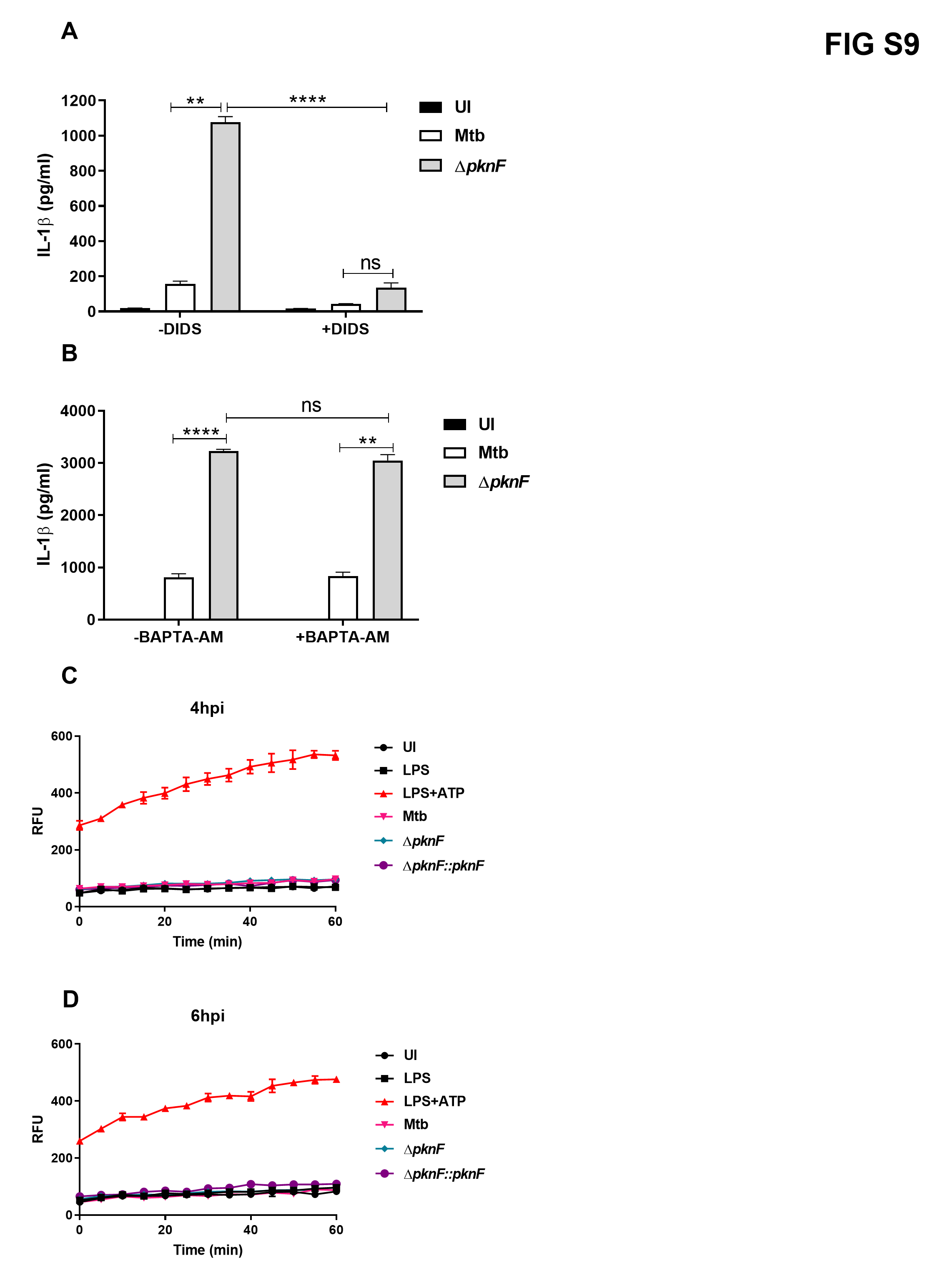

Supplement: S9 Fig — BMDMs were either left untreated or treated with chloride channel blocker, DIDS (100μM) or with BAPTA-AM (30μM), Ca2+ chelator and then infected with CDC1551 Mtb wild-type and ΔpknF mutant. Culture supernatants were harvested at 20 hpi and analyzed for (A, B) IL-1β secretion by ELISA. BMDMs were either left uninfected (UI) or infected with different CDC1551 Mtb strains (Mtb, ΔpknF mutant and complement ΔpknF::pknF) at an MOI of 10 for 4h. BMDMs were primed with LPS (1μg/ml) for 4 h and stimulated with ATP (5mM, 30 min) and used as positive control for inducing NLRP3 inflammasome activation. Intracellular calcium mobilization was analyzed at (C) 4 hpi and (D) 6 hpi by Fluo-Forte Calcium Assay kit. Data are representative of three independent experiments. Error bars represent mean ± SEM; **, p<0.01, ****, p<0.0001, ns (non-significant). (TIF) [file ppat.1009712.s009.tif]

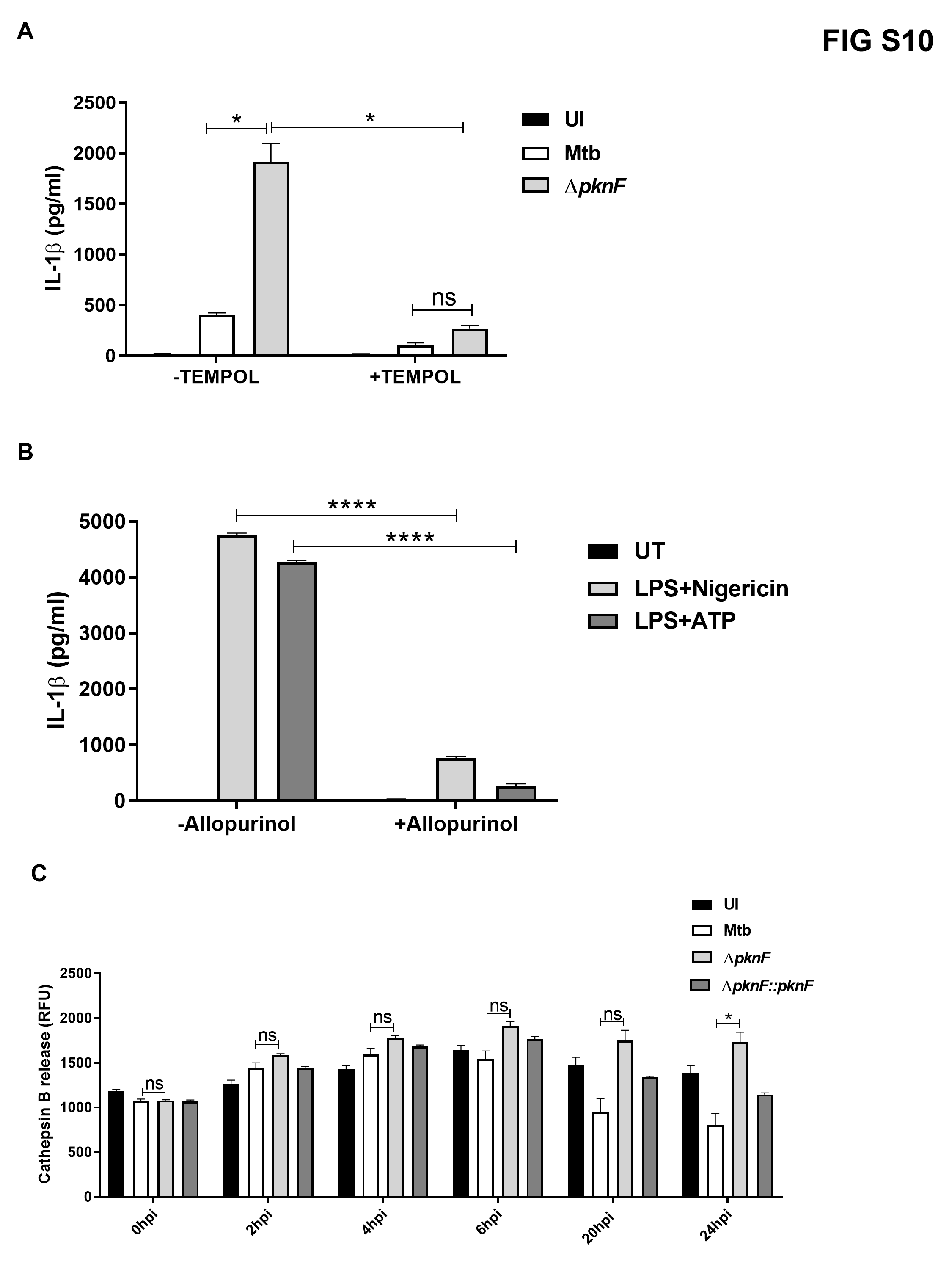

Supplement: S10 Fig — BMDMs were either left untreated (-Tempol) or treated with ROS scavenger, Tempol (100μM) and then infected with CDC1551 Mtb wild-type and ΔpknF mutant at an MOI of 10 for 4h. Culture supernatants were harvested at 20 hpi and analyzed for (A) IL-1β secretion by ELISA. BMDMs were either left untreated (UT) or treated with 1μg/ml LPS for 4 h and then stimulated with two different NLRP3 inflammasome activators, Nigericin (20μM) and ATP (5mM) for 30 min. When required, BMDMs were treated with allopurinol (250μg/ml), xanthine oxidase inhibitor before stimulation with Nigericin/ATP. Cell supernatants were harvested after 30 min of stimulation and analyzed for (B) IL-1β release by ELISA. BMDMs were either left uninfected (UI) or infected with different CDC1551 Mtb strains (Mtb, ΔpknF mutant and complement ΔpknF::pknF). (C) Intracellular cathepsin B activity was monitored at 0, 2, 4, 6, 20 and 24 hpi by Magic Red Cathepsin B assay Kit Data are representative of three independent experiments. Error bars represent mean ± SEM; *, p<0.05, ****, p<0.0001, ns (non-significant). (TIF) [file ppat.1009712.s010.tif]
