# Supplementary material for: Mycobacterium tuberculosis inhibits the NLRP3 inflammasome activation via its phosphokinase PknF
Source: PLoS Pathog. 2021 Jul 29;17(7):e1009712. doi: 10.1371/journal.ppat.1009712 (PMC8321130; doi:10.1371/journal.ppat.1009712)
Supplement: S1 Table — (DOCX) [file ppat.1009712.s011.docx]

**Supporting Information**

**Table S1: Primers used in this study.**

| 1. **Knockout construction**   PknF LF F  PknF LF R  PknF RF F  PknF RF R  **Knockout confirmation**  PknF F  PknF R   1. **Complemented mutant construction**   PknF comp F  PknF comp R | AAGGAGATATCGGGCAACCTGGACTCGGTAGAC  CAGGAAAGCTTGGGGGCGACAAGTGACTCTCCGGTAG  GTAAGGTCTAGAGGGTGGTCGGCTTGCCTGA  ATAATGGTACCGGGCGGGTATCCGGGTGGTG  CTGGACTCGGTAGACGGGGCAGATCTC  GGAACTCCGCACGCGACAAGTGAC  AAGGAATTC ATG CCG CTC GCA GAA GGT TC  AAAAAGCTT TCA TGG CCA GCC GTT GCT TCT |  |  |
| --- | --- | --- | --- |
